# Structural and Functional Analysis of a Platelet-Activating Lysophosphatidylcholine of *Trypanosoma cruzi*

**DOI:** 10.1371/journal.pntd.0003077

**Published:** 2014-08-07

**Authors:** Felipe Gazos-Lopes, Mauricio M. Oliveira, Lucas V. B. Hoelz, Danielle P. Vieira, Alexandre F. Marques, Ernesto S. Nakayasu, Marta T. Gomes, Nasim G. Salloum, Pedro G. Pascutti, Thaïs Souto-Padrón, Robson Q. Monteiro, Angela H. Lopes, Igor C. Almeida

**Affiliations:** 1 The Border Biomedical Research Center, Department of Biological Sciences, University of Texas at El Paso (UTEP), El Paso, Texas, United States of America; 2 Instituto de Microbiologia Paulo de Góes, Universidade Federal do Rio de Janeiro, Cidade Universitária, Centro de Ciências da Saúde, Bloco I, Ilha do Fundão, Rio de Janeiro, Rio de Janeiro, Brazil; 3 Instituto de Biofísica Carlos Chagas Filho, Universidade Federal do Rio de Janeiro, Cidade Universitária, Centro de Ciências da Saúde, Bloco G, Ilha do Fundão, Rio de Janeiro, Rio de Janeiro, Brazil; 4 Universidade Federal de Minas Gerais, Instituto de Ciências Biológicas, Departamento de Parasitologia, Pampulha, Belo Horizonte, Minas Gerais, Brazil; 5 Instituto de Bioquímica Médica, Universidade Federal do Rio de Janeiro, Cidade Universitária, Centro de Ciências da Saúde, Bloco H, Ilha do Fundão, Rio de Janeiro, Rio de Janeiro, Brazil; New York University School of Medicine, United States of America

## Abstract

**Background:**

*Trypanosoma cruzi* is the causative agent of the life-threatening Chagas disease, in which increased platelet aggregation related to myocarditis is observed. Platelet-activating factor (PAF) is a potent intercellular lipid mediator and second messenger that exerts its activity through a PAF-specific receptor (PAFR). Previous data from our group suggested that *T. cruzi* synthesizes a phospholipid with PAF-like activity. The structure of *T. cruzi* PAF-like molecule, however, remains elusive.

**Methodology/Principal findings:**

Here, we have purified and structurally characterized the putative *T. cruzi* PAF-like molecule by electrospray ionization-tandem mass spectrometry (ESI-MS/MS). Our ESI-MS/MS data demonstrated that the *T. cruzi* PAF-like molecule is actually a lysophosphatidylcholine (LPC), namely *sn*-1 C18:1(delta 9)-LPC. Similar to PAF, the platelet-aggregating activity of C18:1-LPC was abrogated by the PAFR antagonist, WEB 2086. Other major LPC species, i.e., C16:0-, C18:0-, and C18:2-LPC, were also characterized in all *T. cruzi* stages. These LPC species, however, failed to induce platelet aggregation. Quantification of *T. cruzi* LPC species by ESI-MS revealed that intracellular amastigote and trypomastigote forms have much higher levels of C18:1-LPC than epimastigote and metacyclic trypomastigote forms. C18:1-LPC was also found to be secreted by the parasite in extracellular vesicles (EV) and an EV-free fraction. A three-dimensional model of PAFR was constructed and a molecular docking study was performed to predict the interactions between the PAFR model and PAF, and each LPC species. Molecular docking data suggested that, contrary to other LPC species analyzed, C18:1-LPC is predicted to interact with the PAFR model in a fashion similar to PAF.

**Conclusions/Significance:**

Taken together, our data indicate that *T. cruzi* synthesizes a bioactive C18:1-LPC, which aggregates platelets via PAFR. We propose that C18:1-LPC might be an important lipid mediator in the progression of Chagas disease and its biosynthesis could eventually be exploited as a potential target for new therapeutic interventions.

## Author Summary

Chagas disease, caused by the parasite *Trypanosoma cruzi*, was exclusively confined to Latin America but it has recently spread to other regions of the world. Chagas disease affects 8–10 million people and kills thousands of them every year. Lysophosphatidylcholine (LPC) is a major bioactive phospholipid of human plasma low-density lipoproteins (LDL). Platelet-activating factor (PAF) is a phospholipid similar to LPC and a potent intercellular mediator. Both PAF and LPC have been reported to act on mammalian cells through PAF receptor (PAFR). Previous data from our group suggested that *T. cruzi* produces a phospholipid with PAF activity. Here, we describe the structural and functional analysis of different species of LPC from *T. cruzi*, including a LPC with a fatty acid chain of 18 carbon atoms and one double bond (C18:1-LPC). We also show that C18:1-LPC is able to induce rabbit platelet aggregation, which is abrogated by a PAFR antagonist. In addition, a three-dimensional model of human PAFR was constructed. Contrary to other *T. cruzi* LPC molecules, C18:1-LPC is predicted to interact with the PAFR model in a fashion similar to PAF. Further studies are needed to validate the biosynthesis of *T. cruzi* C18:1-LPC as a potential drug target in Chagas disease.

## Introduction

*Trypanosoma cruzi* is the etiological agent of Chagas disease, which is associated with myocarditis and vasculitis, accompanied by an increase in inflammatory mediators, such as cytokines, chemokines, phospholipids, and glycolipids [1]–[3]. There is also an increase in platelet aggregation, focal ischemia, and myonecrosis in both acute and chronic stages of the disease [2], [4], [5]. Most of the chronic cases are linked with debilitating cardiomyopathy, which is responsible for more deaths than any other parasitic disease in Latin America [1], [6]. Endemic Chagas disease affects eight to ten million people in 21 countries in Latin America [7]. Chagas disease is also becoming a global health problem because of the migration of unconsciously *T. cruzi*-infected individuals from Latin American countries to other regions of the world. Several thousands of people in the United States, Canada, various European countries, Australia, and Japan are chronically infected with *T. cruzi* [7].

*T. cruzi* has a complex life cycle, with two morphophysiological stages within a triatomine bug and two in a mammalian host. Infectious metacyclic trypomastigotes can be expelled with the insect's excreta during a bloodmeal, reaching the host bloodstream through the bite wound or exposed ocular or oral mucosa. In addition, the insect can acquire the parasite during blood feeding from an infected individual and continue the cycle [8]. Blood transfusion, organ transplantation, congenital transmission and food and fluid contamination are other significant ways of transmitting this disease [9], [10].

In general, lipid mediators [11]–[16] and specifically, lysophosphatidylcholine (LPC) [17], [18], have been implicated in experimental models of Chagas disease. LPC is present in the saliva of at least one of the insect vectors of Chagas disease, the hemipteran *Rhodnius prolixus*, where it acts as an anti-hemostatic molecule and immunomodulator of *T. cruzi* infection in a mammalian model [17]–[19]. LPC (1-acyl-2-hydroxy-*sn*-glycero-3-phosphorylcholine) is a major plasma phospholipid of oxidized low-density lipoproteins (Ox-LDL), albumin and other carrier proteins, being a critical factor in the inflammatory processes and the atherogenic activity of Ox-LDL [20]–[22]. LPC is an intracellular modulator that activates several second messengers, controlling important biological activities, such as cellular proliferation and differentiation, transcription of adhesion molecules and growth factors in endothelial cells, as well as the transportation of fatty acids, choline, and phosphatidylglycerol between tissues [20]–[24]. The biological activities of LPC are usually mediated by G protein-coupled receptors (GPCRs), such as G2A, GPR4, and the receptors for prostacyclin (IP), thromboxane A2 (TXA2) (TP), and platelet-activating factor (PAF) (PAFR) [24]–[33]. Specifically, LPC species are capable of eliciting different cellular activities depending on the length and degree of unsaturation of its sole acyl-chain [30], [34], [35]. Trypanosomatid parasites (e.g., *T. brucei*, *Leishmania* spp., and *T. cruzi*) are known to synthesize phosphatidylcholine (PC) and LPC. Over 50% of the total lipids shed to the culture medium by *T. cruzi* were identified as PC and LPC [36]. These molecules were also found in *Leishmania* [37], [38], African trypanosomes [39], and in the malaria parasite, *Plasmodium falciparum* [40]. To the best of our knowledge, however, the chemical structures of LPC species synthesized by *T. cruzi* have not been defined to date.

Platelet-activating factor (1-*O*-alkyl-2-acetyl-*sn*-glycero-3-phosphocholine; PAF) is structurally very similar to LPC [41]. PAF exhibits potent biological activity and is synthesized by a wide variety of cells, including neutrophils, platelets, macrophages, and lymphocytes [42]. PAF induces numerous physiological and pathophysiological effects, such as cellular differentiation, inflammation, and allergy, through the activation of specific GPCRs with seven transmembrane helices [25], [43]. We have previously shown that *T. cruzi* synthesizes a lipid with platelet-aggregating properties similar to PAF [14]. Preliminary structural analysis by chemical and enzymatic treatment indicated that the *T. cruzi* PAF-like lipid, metabolically labeled with ^14^C-acetate, was labile to mild-alkaline or hydrofluoric acid hydrolysis, suggesting a molecule containing a glycerolipid moiety with at least one acyl chain and a phosphate group [14]. However, the detailed structure of the *T. cruzi* PAF-like lipid remains elusive.

Here, we describe the identification and bioactivity of the as-yet elusive *T. cruzi* PAF-like molecule. We use a novel approach for the enrichment of this and other closely related lysophospholipids, followed by tandem mass spectrometry (MS^n^) to provide ample structural information. Moreover, we constructed a 3-D molecular model of PAFR and used molecular docking to predict the interactions of the *T. cruzi* PAF-like molecule and other lysophospholipids with the receptor.

## Materials and Methods

### Ethics statement

Rabbit platelets used in this study were obtained following the guidelines of the Committee for Evaluation of Animal Use for Research of the Federal University of Rio de Janeiro (CAUAP-UFRJ) and the NIH Guide for the Care and Use of Laboratory Animals. The vertebrate animal protocol was approved by CAUAP-UFRJ under registry number IBQM011.

### Chemicals

Synthetic C16:0-, C18:0-, C18:1(Δ^9^)-, and C22:6-LPC, and C16:0-PAF were purchased from Avanti Polar Lipids (Alabaster, AL). The competitive PAF antagonist WEB 2086 (4-[3-[4-(2-chlorophenyl)-9-methyl-6h-thieno[3,2-f][1], [2], [4]triazolo[4,3-a]diazepin-2-yl]-1-oxopropyl]morpholine) was kindly provided by Dr. H. Heurer from Boehringer Ingelheim (Ingelheim, Germany). Otherwise indicated, all other reagents and solvents used here were of analytical, HPLC, or mass spectrometric grade from Sigma-Aldrich (St. Louis, MO).

### *Trypanosoma cruzi* culture

All *T. cruzi* life cycle stages or forms were obtained from the Y strain [44]. Epimastigote forms (Epis) were maintained by weekly transfers using liver infusion tryptose (LIT) medium [45], supplemented with 0.002% hemin and 10% heat-inactivated fetal calf serum (FCS; Hyclone, heat-inactivated at 56°C for 30 min) at 28°C. Metacyclic trypomastigote forms (Metas) were obtained by spontaneous axenic differentiation of Epis at 28°C, followed by their purification using ion-exchange chromatography [46], [47]. Mammalian tissue culture-derived trypomastigotes (TCTs) were obtained from the supernatants of 5 to 6 days old *T. cruzi*-infected LLC-MK2 cells (American Type Culture Collection, Rockville, MD), maintained in RPMI-1640 medium supplemented with 2% FCS at 37°C in a 5% humidified CO_2_ atmosphere [48]. Intracellular amastigotes (ICAs) were obtained as described [49]. Briefly, infected monolayers of LLC-MK2 cells were gently detached by scraping (BD Falcon cell scraper, BD Biosciences) and resuspended in PBS supplemented with 10% FCS. Mammalian cells were disrupted by passage through a 27-gauge needle (BD, Becton and Dickinson & Co.). ICA forms were separated from the cell debris by centrifugation (800× *g* for 5 min at 4°C). The supernatant was then harvested and passed through a DE-52 column and parasites were incubated for 2 h at 37°C in a humidified 5% CO_2_ atmosphere, after which the parasites were again passed through a DE-52 column, from which they were harvested and stored. For the viability testing of all parasite forms, cells were resuspended in a Trypan Blue solution and counted in a Neubauer chamber [50]. In this study, all experiments were performed using parasites that were harvested by centrifugation and washed three times with PBS before use, unless otherwise specified. All parasite forms were counted and then frozen in liquid nitrogen prior to use.

### Lipid extraction

Frozen pellets derived from Epis, Metas, ICAs, and TCTs (2×10^9^ cells each), were suspended in 1.6-ml ice-cold HPLC-grade water and transferred to 13×100-mm Pyrex culture tubes with polytetrafluoroethylene (PTFE)-lined screw caps. HPLC-grade chloroform and methanol were added to each vial, giving a final ratio of chloroform/methanol/water (C/M/W) of 1∶2∶0.8 (v/v/v). The samples were mixed vigorously using a vortex for 2 min and then centrifuged for 15 min at 1,800× *g* at room temperature. After centrifugation, the supernatants were transferred to PTFE-lined Pyrex glass test tubes and the pellets were dried under a constant flow of N_2_ stream. The dry pellets were then extracted three times with C/M (2∶1, v/v) and twice with C/M/W (1∶2∶0.8, v/v/v). After extraction, the supernatants were pooled together and dried before being subjected to Folch's partition [51]. To this end, samples were first dissolved in C/M/W (4∶2∶1.5, v/v/v) and then mixed vigorously for 5 min using a vortex and finally centrifuged for 15 min at 1,800× *g* at room temperature. After centrifugation, the lower (organic) and upper (aqueous) phases were separated in PTFE-lined Pyrex glass test tubes. The Folch lower phase was then washed two times with a freshly prepared upper phase, dried under N_2_ steam, and stored at −70°C until use.

### Solid-phase extraction (SPE) of phospholipids

Phospholipids derived from the lower phase of the Folch's partition, as described above, were purified from other classes of lipids using a three-step SPE protocol [52]. Briefly, 100 mg silica gel (Merck, grade 7754, high purity, 70–230 mesh, 60 Å) were packed into borosilicate glass Pasteur pipettes (5 ¾″, Fisher Scientific) using Pyrex glass fiber wool (8-µm pore size, Sigma-Aldrich) as a sieve. The column was sequentially conditioned with 4 ml each methanol, acetone, and chloroform. Dried Folch lower phase samples from all *T. cruzi* life stages were redisolved in 3 ml chloroform and a third of each sample was added to the column. Lipids were sequentially eluted with 4 ml chloroform (neutral lipids), acetone (glycolipids), and methanol (phospholipids and free fatty acids). Each fraction was collected into a 7-ml amber glass vial with PTFE-lined screw top (SUPELCO, Sigma-Aldrich). All samples were immediately dried under a constant flow of N_2_ stream and stored at −70°C until use.

### *T. cruzi* PAF-like phospholipid enrichment

With the purpose of enriching the putative PAF-like molecule from a complex *T. cruzi* phospholipid mixture, a novel method was developed using perfusion chromatography [53]. Briefly, fifty microliters of a suspension of 40 mg/ml POROS R1 50 (poly[styrene/divinylbenzene], with similar binding strength as C4 supports) beads (Applied Biosystems, 50-µm diameter) in HPLC-grade n-propanol (Honeywell, Burdick & Jackson, Radnor, PA) were packed into a 200-µl sterile micropipette tip (Axygen, Corning Life Sciences, Union City, CA). Pyrex glass fiber wool (8-µm pore size, Sigma-Aldrich) was used as sieve. The POROS R1 mini-column was washed twice with 100 µl HPLC-grade methanol and then conditioned with an n-propanol/water gradient (from 50% to 0%, in 5% increments). Each step of the gradient was performed with 100 µl solvent, with the exception of the last step (0% n-propanol), which was performed twice with 100 µl HPLC-grade water. Then, either the mixture of lipid standards (100 pmol C16:0-*lyso*-PC (C16:0-LPC), C16:0-*lyso*-PAF (C16:0-LPAF), C16:0-PAF, and C18:0/C18:1-diacyl-PC) or *T. cruzi* phospholipids derived from the SPE procedure were suspended in HPLC-grade water, sonicated for 10 min in a bath sonicator, and added to the column. Both the standards and the phospholipid extracts were eluted using a 0%–50% *n*-propanol gradient in 5% *n*-propanol increments. Each fraction was stored in Axygen 2-ml microcentrifuge tube at −70°C until further use.

### Structural characterization of LPC species by tandem electrospray ionization-linear ion trap-mass spectrometry (ESI-LIT-MS)

All fractions derived from the POROS R1 mini-column purification, as well as the fractions obtained prior to this last procedure (namely, the lower Folch and methanol phase), were dissolved in MS-grade methanol containing 5 mM LiOH, as indicated. Samples were directly injected (at 300 nl/min) by chip-based infusion using a TriVersa NanoMate nanoelectrospray source (Advion, Ithaca, NY), into an LTQXL ESI-linear ion trap-MS (ESI-LIT-MS) (Thermo Fisher Scientific), in positive-ion mode. The source voltage was set at 0.01 kV and current at 0.03 µA; capillary voltage and temperature were 36 V and 150°C, respectively; and tube lens voltage was set at 145 V. Select ions were subjected to sequential tandem fragmentation (MS^n^) by collision-induced dissociation (CID). Full-scan (MS) spectra were collected at the 400–1000 *m/z* range. Tandem mass fragmentation was carried out using normalized collision energies of 35, 40, and 45 for MS^2^, MS^3^, and MS^4^, respectively. The resulting spectra were compared to the aforementioned phospholipid standards, as well as to previously described results.

The location of the acyl chain on *T. cruzi* LPC species was determined by MS^2^ and M^3^ analysis, essentially as described by Hsu et al. [54]. Briefly, *sn*-1 and *sn*-2 C18:1-LPC regioisomer standards were generated by treatment of 18:1(Δ9-*cis*)-PC (1,2-dioleoyl-*sn*-glycero-3-phosphocholine, catalog # 850375, Avanti Polar Lipids) with either PLA2 (from porcine pancreas, catalog # P6534, Sigma-Aldrich) or PLA1 (from *Thermomyces lanuginosus*, catalog # L3295, Sigma-Aldrich). For PLA1 treatment, one mg of diacyl-PC standards were dried under N2 stream, redisolved in 200 µL of reaction buffer (50 mM Tris-HCl, 2 mM CaCl_2_, 140 mM NaCl, pH 8.0) and sonicated for 30 min. Afterwards, the samples were incubated at 37°C for 2 h in the presence of 12 units PLA1. The reaction was interrupted by the addition of 1.5 mL chloroform, followed by vortexing for 1 min. The resulting LPC species present in the organic phase of the mixtures were then purified by SPE, following the protocol described above. Finally, LPCs were recovered in the methanolic phase of the SPE column. PLA2 treatment was performed following the same procedure steps used for PLA1 treatment but using a different reaction buffer (100 mM Tris-HCl, 5 mM CaCl_2_, 100 mM NaCl, pH 8.0) and 5 units PLA2 [55]. The purified lipids (methanolic phase of the SPE) were analyzed by ESI-LIT-MS in 100% methanol containing 5 mM LiOH or 5 mM NaCl. The MS analyses were performed as described above. The assignment of the fatty acid position on the LPC (*sn*-1 or *sn*-2) was performed by comparing the fragmentation pattern of standard *sn*-1 and *sn*-2 C18:1-LPCs with *T. cruzi*-derived 18:1-LPC samples.

The position of the double bond on fatty acid substituents was determined by MS4, essentially as described by Hsu et al. [56]. Briefly, *sn*-1 C18:1(Δ6-*cis*)-LPC and sn-1 C18:1(Δ9-*cis*)-LPC standards were generated by treatment of both 18:1(Δ6-*cis*)-PC (1,2-dipetroselenoyl-*sn*-glycero-3-phosphocholine, catalog # 850374, Avanti Polar Lipids) and 18:1(Δ9-*ci*s)-PC (1,2-dioleoyl-*sn*-glycero-3-phosphocholine, catalog # 850375, Avanti Polar Lipids) with PLA2 (from porcine pancreas, catalog # P6534, Sigma-Aldrich), as described above. LPC species were recovered from incubation mixtures and analyzed by ESI-LIT-MS in 100% methanol containing 5 mM LiOH, as described above.

### Quantification of *T. cruzi* LPC species

Commercial C10:0-LPC (1-decanoyl-2-hydroxy-*sn*-glycero-3-phosphocholine, catalog # 855375, Avanti Polar Lipids) was used as an internal standard to quantify the most abundant LPC species found in *T. cruzi* samples. Briefly, 18 nmoles of standard C10:0-LPC were added to parasite pellets (7×10^8^ cells) shortly before lipid extraction. Lipid extraction was conducted on freshly prepared parasite pellets following the protocol described above. Then, the Folch lower-phase fractions were analyzed by ESI-MS (at the 400–1000 *m/z* range) under the identical MS conditions used for the characterization of *T. cruzi* LPCs. The amount of each LPC species was calculated using the formula: [*T. cruzi* LPC peak intensity/C10:0-LPC peak intensity]×[concentration of C10:0-LPC (18 pmol/µl)/MRRF], where, MRRF stands for the molar relative response factor of each LPC species to the C10:0-LPC standard. The MRRF was calculated by dividing the intensity of the peak corresponding to a standard LPC (C16:0-LPC, C18:0-LPC, or C18:1-LPC) by the intensity of the peak corresponding to C10:0-LPC (*m/z* 418.5), when all molecules were in equimolar concentrations.

*T. cruzi* LPC species were also quantified in extracellular vesicles (EV) and EV-free supernatant or conditioned medium (VF) of Epis (eV2, eV16, and eVF) and Metas (mV2, mV16, and mVF), obtained from Epi and Meta pellets (9×10^9^ parasites each), as previously described [57]. C10:0-LPC (*m/z* 418) (18 nmoles per sample) was used as an internal standard. LPC species were extracted from Epi- and Meta-derived EVs, and respective total parasite pellets (ePellet and mPellet) as described above. The Folch lower phase fractions were analyzed by ESI-LIT-MS as above.

### Platelet aggregation assay

C16:0-PAF and various synthetic (C16:0-, C18:0-, C18:1-, and C22:6-LPC) or purified (C18:2-LPC) LPC species were tested in a platelet aggregation assay [58]. Rabbit blood platelets were prepared from blood collected with 5 mM EDTA as anticoagulant, isolated by centrifugation, washed and resuspended in a modified Tyrode's buffer, pH 7.4, containing 2 mM CaCl_2_, at a final concentration of 3–4×10^5^ cells/µl in Tyrode's buffer. Platelet aggregation experiments were performed with a Chronolog Aggregometer (Havertown, PA, USA), with monitoring time of 5 min. Rabbit platelets used in this investigation were obtained following the guidelines for animal experimentation of the USA National Institutes of Health and the experimental protocol received official approval of the Institutional Animal Care and Use Committee, Universidade Federal do Rio de Janeiro.

### Comparative modeling

The amino acid sequence of the human PAF receptor (PAFR, UniProtKB ID: P25105) was obtained from ExPASy server [59]. The region between Asp10-Ser310, part of PAFR sequence that includes all seven-transmembrane domains, was submitted to I-TASSER server, which combines threading and *ab initio* algorithms [60], [61]. The I-TASSER server, ranked as the best server in recent CASP7 and CASP8 experiments, builds protein models based on multiple-threading alignments by LOMETS program and iterative TASSER program simulations [61], [62]. In addition, MODELLER v9.10 program [63], [64] (http://salilab.org/modeller/) was used to add a disulfide bridge between Cys90-Cys173 and, subsequently, to refine the best I-TASSER model. Thus, the final model was validated using three programs: PROCHECK [65] and ERRAT [66], both at SAVES server (http://nihserver.mbi.ucla.edu/SAVES_3/) and PROQM [67] (available as a server at http://www.bioinfo.ifm.liu.se/services/ProQM/index.php?about=proqm). PROCHECK analyzes the stereochemical quality and ERRAT evaluates the non-bonded atomic interactions in the model structure, while PROQM uses a specific-scoring function for membrane protein, including GPCR, to assess local and global structural quality of the model.

### Molecular docking

The ligand structures (C16:0-PAF, C16:0-LPC, C18:0-LPC, C18:1-LPC, and C18:2-LPC) were built in the Spartan'10 software (Wavefunction, Inc., Irvine, CA). The docking of the ligands to the PAFR model binding site was performed using Molegro Virtual Docker (MVD) program (CLC bio, Aarhus, Denmark), which uses a heuristic search algorithm that combines differential evolution with a cavity prediction algorithm. The MolDock scoring function used is based on a modified piecewise linear potential (PLP) with new hydrogen bonding and electrostatic terms included. Full description of the algorithm and its reliability compared to other common docking algorithm have been described [68]. As no satisfactory cavities were found by cavity prediction algorithm using MVD, His248 (a constituent residue of the binding pocket) was set as center of searching space. The search algorithm MolDock optimizer was used with a minimum of 50 runs and the parameter settings were: population size = 200; maximum iteration = 2000; scaling factor = 0.50; offspring scheme = scheme 1; termination scheme = variance-based; crossover rate = 0.90. Due to the stochastic nature of algorithm search, ten independent simulations per ligand were performed to predict the binding mode. Consequently, the complexes with the lowest interaction energy were evaluated. The interactions between PAFR and each ligand were analyzed using the ligand map algorithm, a standard algorithm in MVD program. The usual threshold values for hydrogen bonds and steric interactions were used. All figures of PAFR modeling and docking were edited using Visual Molecular Dynamics (VMD) program (available for download at http://www.ks.uiuc.edu/Research/vmd/vmd-1.9.1/).

## Results

### Enrichment of LPC and PAF from complex mixtures

**Figure 1:**
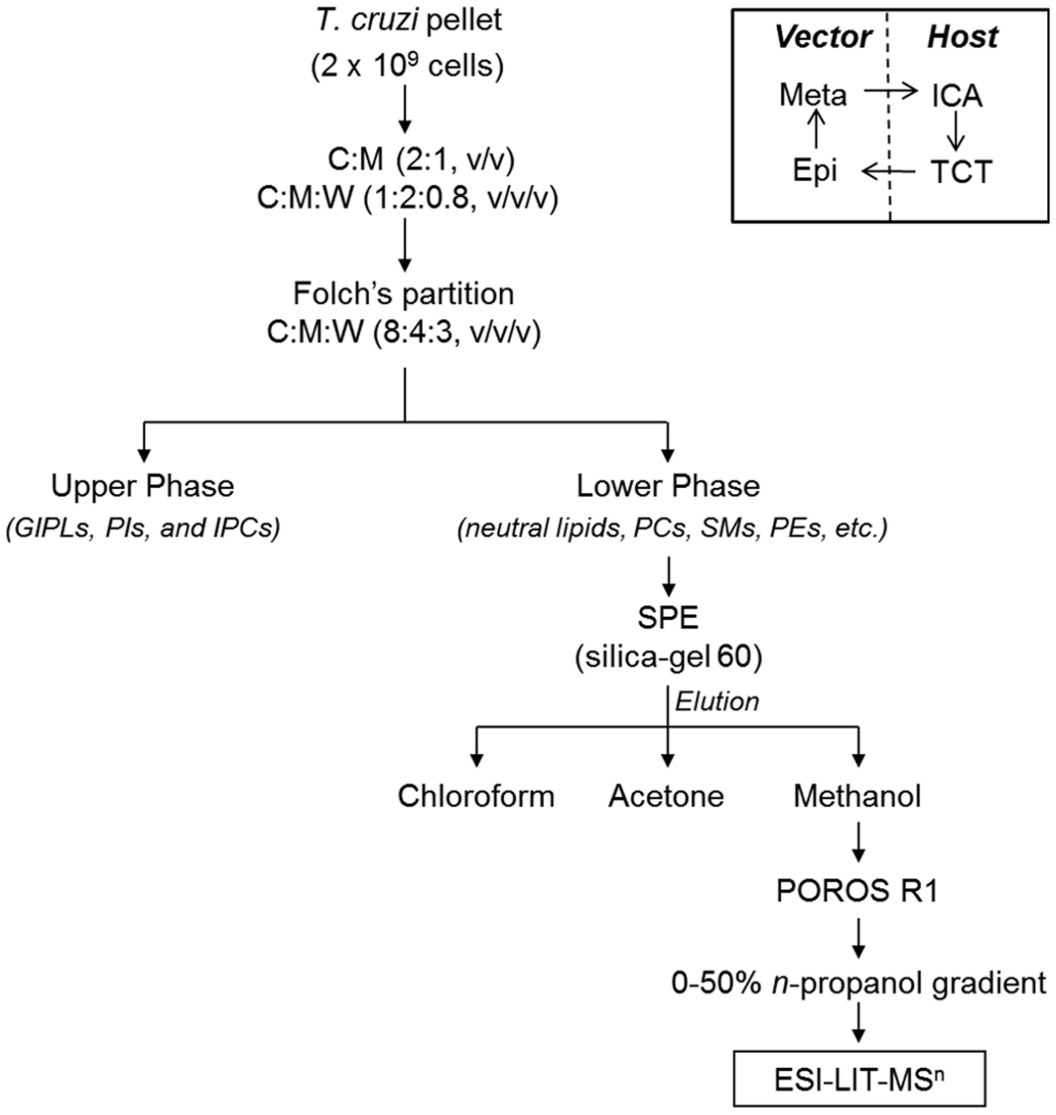
Schematic representation of the methodology used for the enrichment and analysis of the putative *T. cruzi* PAF-like phospholipid. The total lipid content of the pellets from *T. cruzi* epimastigote (Epi), metacyclic trypomastigote (Meta), tissue culture-derived trypomastigote (TCT), or intracellular amastigote (ICA) forms was extracted with organic solvents followed by Foch's partition. Folch lower-phase samples were fractionated by SPE in a silica gel (60 Å) column. The different lipid classes were eluted with chloroform (neutral lipids), acetone (glycolipids), and methanol (phospholipids). The latter was further fractionated by perfusion chromatography using POROS R1 50 mini-columns, eluted with a 0%–50% *n*-propanol gradient. All fractions were diluted in methanol containing 5 mM LiOH and analyzed by MS^n^. The inset depicts the *T. cruzi* life cycle with the four stages used in this study.

**Figure 2:**
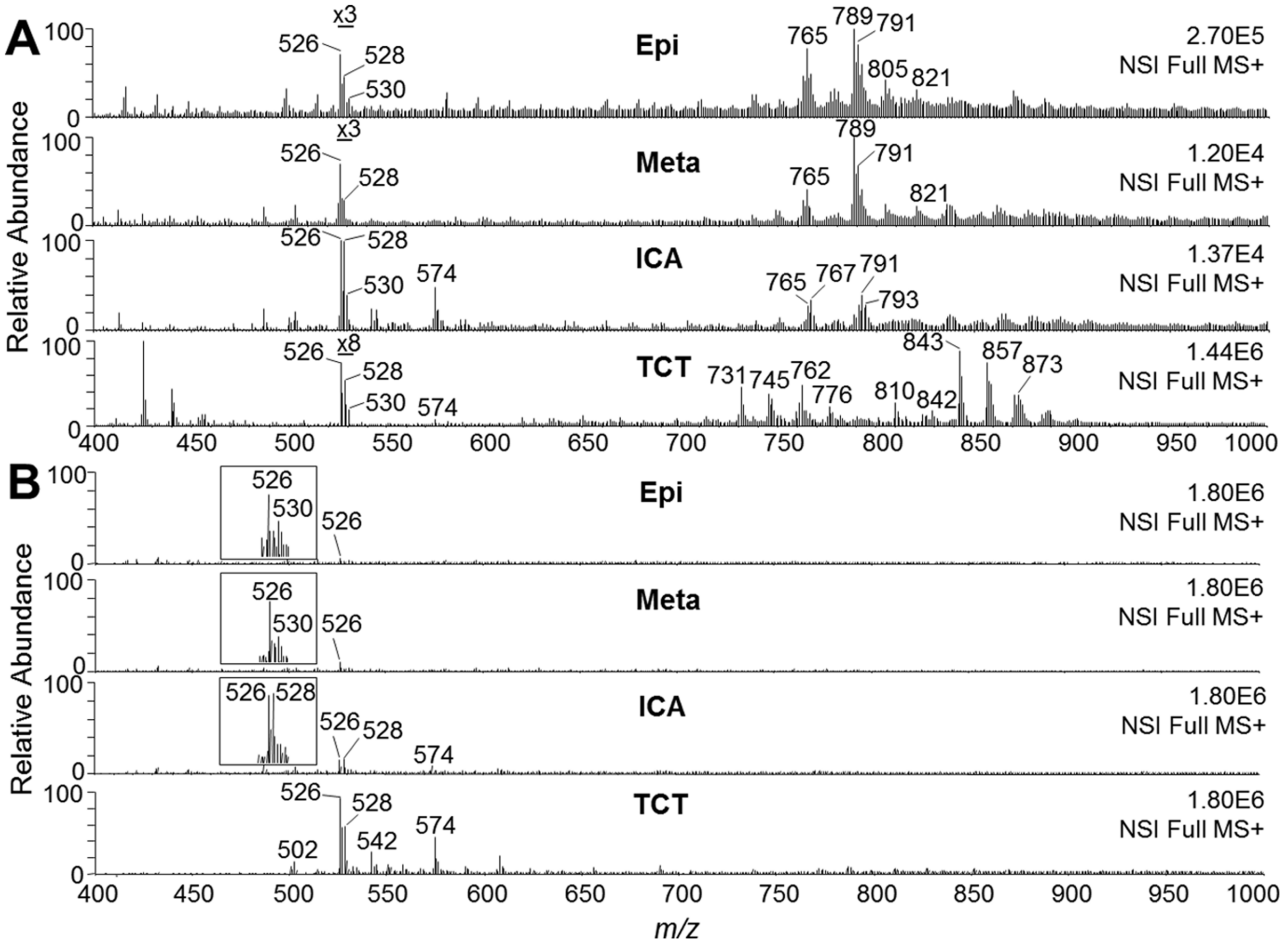
Full ESI-LIT-MS spectra of *T. cruzi* phospholipids. (**A**) MS1 spectra of lipids obtained in the Folch lower phase prior to fractionation. Lipid samples from all *T. cruzi* stages were diluted in methanol containing 5 mM LiOH and analyzed by direct infusion in an LTQXL ESI-LIT-MS (positive-ion mode, MS+). Note that the region of spectrum corresponding to LPAF, LPC, and PAF species in Epi, Meta, and TCT has been magnified for better visualization. (**B**) MS1 spectra of phospholipids obtained by SPE followed by POROS R1 fractionation. Lipids eluted in 25% n-propanol were diluted in methanol containing 5 mM LiOH and analyzed as above. Since the same initial total number of cells (5×10^8^) was used for lipid fractionation from each parasite stage, all spectra were normalized. Magnification of the MS range where PAF and LPC species would be found is indicated (insets). Epi, epimastigote; Meta, metacyclic trypomastigote; ICA, intracellular amastigote; TCT, tissue culture-derived trypomastigote. *m/z*, mass to charge ratio.

Previous results from our group strongly indicated that *T. cruzi* synthesizes a phospholipid with platelet-aggregating activity similar to PAF [14]. Thus far, however, the precise structure of this bioactive parasite-derived molecule remains unknown. Aiming at the enrichment and characterization of the putative *T. cruzi* PAF-like phospholipid from a complex phospholipid mixture, we developed a fractionation protocol, which included solvent extraction and Folch's partition, followed by SPE and perfusion chromatography (Fig. 1). Lipid fractions obtained after each step of purification were analyzed by ESI-LIT-MS in positive-ion mode. Total-ion mapping (TIM) for the neutral loss of the trimethylamine group ( = 59 a.m.u.) was performed to promptly localize phosphocholine-containing phospholipids of all life-cycle stages of *T. cruzi* (data not shown). ESI-LIT-MS analysis of the Folch lower phase of the four parasite forms (Epi, Meta, ICA, and TCT) showed major phospholipid species at the 700–900 *m/z* range, except for the ICA form (Fig. 2A). Tandem MS (MS^n^) analysis of these lipid species revealed that, as expected, they were mostly diacyl-PC and sphingomyelin (SM) species (data not shown, to be published elsewhere). In contrast to other parasite forms, ICA is much richer in lipid species at the 500–600 *m/z* range, particularly *m/z* 526, 528, 530, and 574 (Fig. 2A). Noteworthy, lithiated singly-charged ion species ([M+Li]^+^) of synthetic LPAF, LPC, and PAF standards were found at this range of the spectrum (Fig. S1, top spectrum). ESI-LIT-MS analysis of SPE-derived fractions of all *T. cruzi* forms revealed a clear enrichment of phosphocholine-containing lipids at the low *m/z* range (400–600), which would indicate an enrichment of potential LPC, LPAF, or PAF molecules. Nevertheless, most samples still contained high amounts of diacyl-PCs and, possibly, SMs (data not shown). Therefore, a novel protocol using POROS R1 beads was designed to enrich the putative PAF-like molecule, which as we predicted could have a structure similar to PAF, LPAF, or LPC. First, we tested the POROS R1 mini-column with a complex mixture of phospholipid standards containing LPAF, LPC, PAF, and diacyl-PCs. We were able to obtain highly enriched LPAF, LPC, and PAF species in the 20% and 25% n-propanol fractions (Fig. S1). Identical conditions were applied for further fractionation of the phospholipids present in the SPE methanolic fraction of all *T. cruzi* forms. The 25%-n-propanol fractions from these parasite stages were then compared by positive-ion mode ESI-LIT-MS, using the same concentration of cells (4×10^5^/µl) and flow rate (300 nl/min) (Fig. 2B). The ion species at the 700–900 *m/z* range, corresponding to diacyl-PCs and SMs, were noticeably much less abundant in the 25%-*n*-propanol POROS R1 fraction (Fig. 2B) than in the Folch lower phase and SPE methanolic fractions (Fig. 2A and data not shown), which is in agreement with the observed phospholipid standard results (Fig. S1). In contrast, the ion species at the 400–600 *m/z* range were prominently more abundant in the 25%-*n*-propanol POROS R1 fraction than in lower Folch and SPE methanolic fractions (Fig. 2A,B and data not shown). In particular, the ion species at *m/z* 526 and 528 were remarkably abundant in the infective TCT form and in the noninfective ICA form. Tandem MS was then performed for the elucidation of the molecular structures of all phosphocholine-containing lysophospholipids at the 400–600 *m/z* range.

### Structural characterization and quantification of *T. cruzi* lysophospholipid species by tandem MS

**Figure 3:**
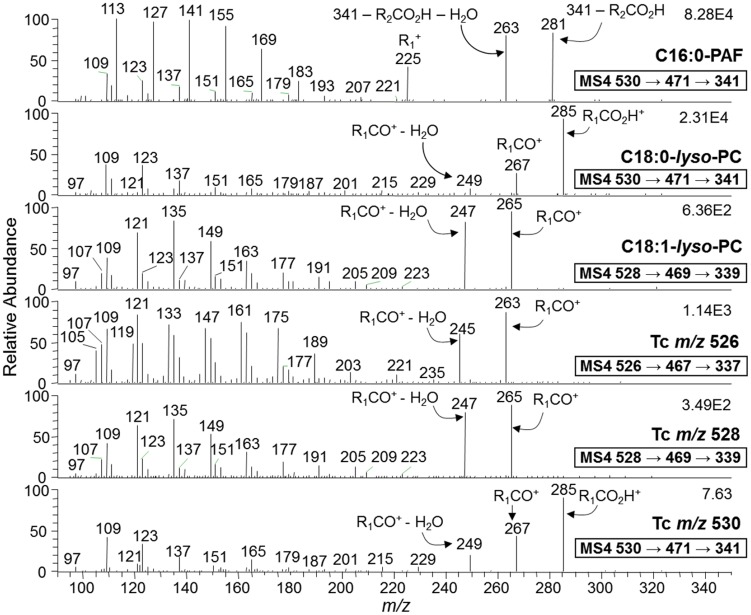
ESI-LIT-MS^4^ analysis of major *T. cruzi* ion species enriched by POROS R1 fractionation. Phospholipid standards (C16:0-PAF, C18:0-LPC, and C18:1-LPC) or *T. cruzi* phospholipids from the POROS R1 25% *n*-propanol fractions were diluted in methanol containing 5 mM LiOH and then infused directly into the LTQXL MS. Fragment ion species resulting from neutral loss of 59 *m/z* (trimethylamine) from the parent ion at MS^2^ were selected for MS^3^ fragmentation (Suppl. Figs. 2A and 2B). Then, ion species resulting from neutral loss of 189 *m/z* (phosphocholine+Li) from parent ions observed at MS^3^ were selected for MS^4^ fragmentation. All spectra shown were obtained from TCT preparation. Identical fragmentation patterns were observed from Epi, Meta, and ICA samples.

**Figure 4:**
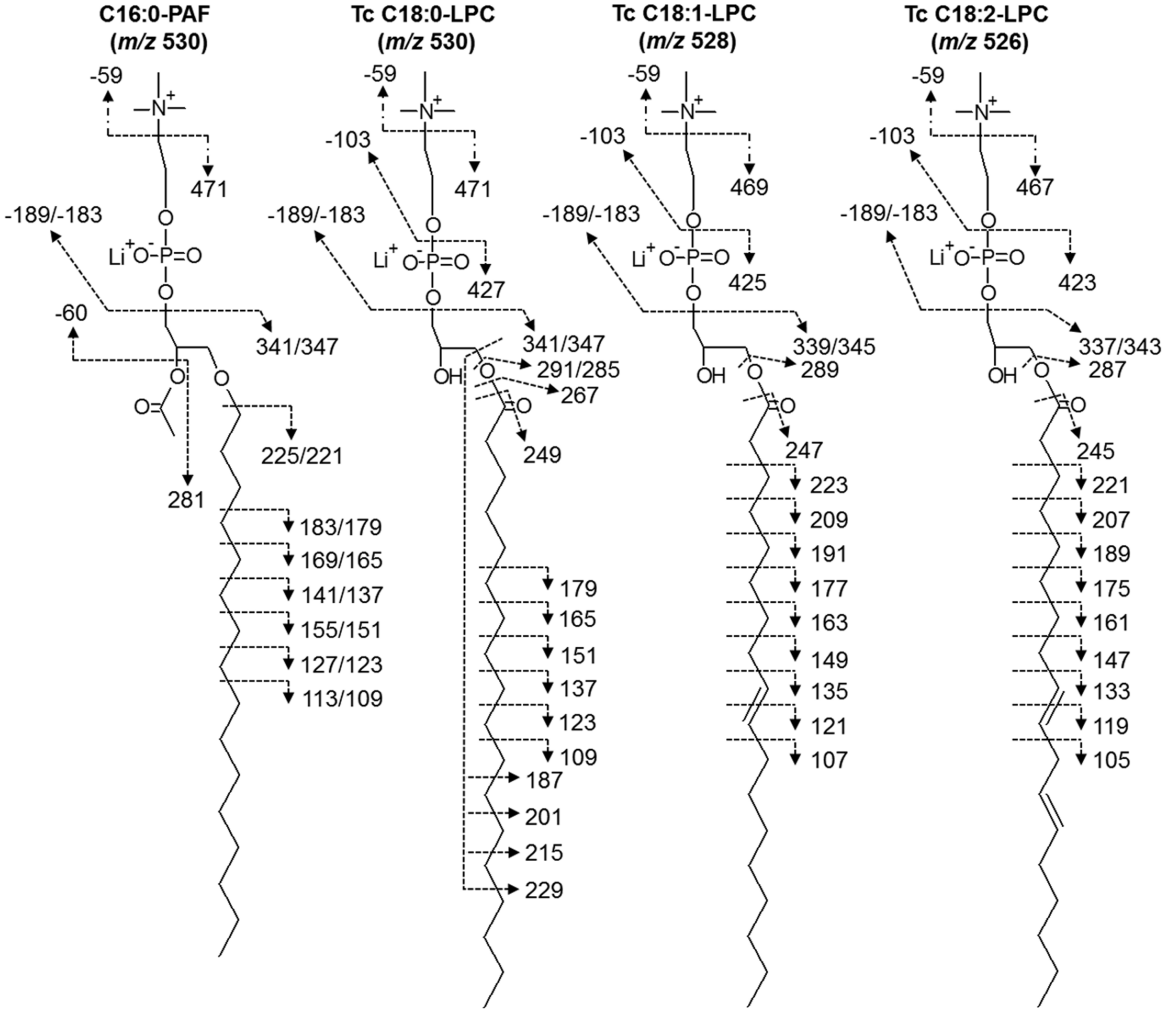
Proposed molecular structures of the three major LPC species of *T. cruzi*. Fragment ions obtained from the MS^n^ analysis of *T. cruzi* ion species at *m/z* 530, 528, and 526 are indicated. The tandem fragmentation of C16:0-PAF standard is included as a reference.

The fragmentation pattern of the synthetic C16:0-PAF standard (*m/z* 530) was compared to those of synthetic C18:0- and C18:1-LPC standards (*m/z* 530 and 528, respectively), because certain PAF and LPC species may be isobaric. The assignment of the lysophospholipid species found in the 25%-*n*-propanol POROS R1 fraction of all *T. cruzi* forms was based on the fragmentation pattern of these standards, as well as on previously reported results [41], [54], [56]. Tandem MS (MS^2^) analysis of singly-charged, lithiated C16:0-PAF, C18:0-LPC, and C18:1-LPC ion species gave rise to fragment ions at *m/z* 471, 471, and 469, respectively, corresponding to the neutral loss of 59 a.m.u. ( = trimethylamine group). The fragmentation of 16:0-PAF standard, however, also gave rise to a fragment ion at *m/z* 341, consistent with the neutral loss of the whole phosphocholine headgroup along with the lithium adduct (−189 a.m.u.). This fragment could not be detected on either LPC standards (Fig. S2A). MS^3^ Fragmentation of the major ions obtained by MS^2^ of C16:0-PAF, C18:0-LPC, and C18:1-LPC (i.e., *m/z* 471, 471, and 469), gave rise to the major non-lithiated ion fragments at *m/z* 341, 341, and 339, respectively, corresponding to the loss of 130 a.m.u. ( = ethyl phosphate group+Li) (Fig. S2B). In addition, we observed lithiated ion fragments at *m/z* 347, 347, and 345, corresponding to the loss of 124 a.m.u. ( = ethyl phosphate group), for C16:0-PAF, C18:0-LPC, and C18:1-LPC, respectively. Interestingly, C18:0-LPC and C18:1-LPC also gave rise to two fragment ions (*m/z* 291 and 289, respectively) that could not be detected in the C16:0-PAF standard. These fragment ions corresponded to the lithiated ([R_1_CO_2_H+Li]^+^) stearoyl (*m*/z 291) and oleyl chains (*m*/z 289), after the loss of choline (N^+^(CH_3_)_3_(CH_2_)_2_OH) from the precursor ions *m/z* 427 and 425, respectively (Fig. S2B) [54]. Finally, we carried out MS^4^ analysis of the major fragment ion species obtained by MS^3^ of C16:0-PAF, C18:0-LPC, and C18:1-LPC standards (*m/z* 341, 341, and 339, respectively) (Fig. 3). A complex fragmentation pattern that provided ample structural information for all three standards was observed. In these spectra, it was possible to identify fragment ions generated by the loss of the acetyl group at the *sn*-2 position of C16:0-PAF (*m/z* 281 and 263), and a fragment ion corresponding to the protonated C16:0-alkyl chain (R_1_^+^) (*m/z* 225). Moreover, a series of fragments resulting from the loss of methylene groups ( = 14 a.m.u.) from the PAF C16:0-alkyl chain could also be seen below *m/z* 225. Similar information could be obtained from the C18:0-LPC and C18:1-LPC, where fragment ions derived from the protonated stearoyl (*m/z* 285, 267, and 249) and oleyl (*m/z* 265 and 247) acyl chains at the *sn*-1 position could be identified [54]. In both cases, a series of fragments corresponding to the loss of methylene units could also be seen below *m/z* 240 (Fig. 3). Based on the fragmentation pattern of the standards in MS^2^, MS^3^, and MS^4^, we could assign the different lysophospholipid species of *T. cruzi* enriched in the POROS R1 25%-n-propanol fraction. As observed in Figs. 3 and S2A,B, the parent ions at *m/z* 526, 528, and 530 corresponded to C18:2-LPC, C18:1-LPC, and C18:0-LPC, respectively. The lithiated ([R_1_CO_2_H+Li]^+^), non-lithiated ([R_1_CO^+^]^+^, and dehydrated [R_1_CO^+^ - H_2_O]^+^) fragment ions of linoleyl (*m*/z 287, 263, and 245), oleyl (*m*/z 289, 265, and 247), and stearoyl (*m*/z 291, 285, 267, and 249) chains, respectively, corroborated our assignments of *T. cruzi* (Tc) *m/z* 526, 528, and 530 as C18:2-, C18:1, and C18:0-LPC (Figs. 3 and S2A,B). No detectable traces of C16:0-PAF (isobaric to C18:0 LPC) or any other PAF-like species could be found. The structure and the fragmentation pattern of C16:0-PAF and the major *T. cruzi* LPC species are represented in Fig. 4.

The MS^n^ experiments carried out above, however, could not provide sufficient structural information to determine the position of the fatty acid (*sn*-1 or *sn*-2) and the location of the double bonds on three major LPCs of *T. cruzi*. To address this point, we first generated LPC standards with fatty acids on either the *sn*-1 or *sn*-2 position by treating diacyl-PCs with commercial PLA2 and PLA1, respectively. Following protocols by Hsu et al. [54], we were able to determine that the acyl chain in the three major *T. cruzi* LPCs was localized at the *sn*-1 position. In Figs. S3A,B, the fragmentation spectra (MS^2^ and MS^3^) of sodiated and lithiated *sn*-1 C18:1-LPC, *sn*-2 C18:1-LPC, and *T. cruzi* C18:1-LPC (from ICA form) are shown. In agreement with Hsu et al. [54], the relative abundance of the sodiated parent ion (*m/z* 544) to the fragment ion (*m/z* 485), corresponding to the loss of trimethylamine (−59 a.m.u), could be used to differentiate between the two possible regioisomers (Fig. S3A). Clearly, *T. cruzi* C18:1-LPC showed a fragmentation pattern consistent with an acyl chain located at *sn*-1. This result was corroborated by the fragmentation spectrum of the lithiated *T. cruzi* C18:1-LPC (Fig. S3B). In this case, the relative abundance of the fragment ion at *m/z* 425 [M – N^+^(CH_3_)_3_(CH_2_)_2_OH+Li^+^]^+^ to the ions at *m/z* 339 (M - 189) and *m/z* 345 (M - 183) was used to corroborate the *sn*-1 position of the acyl chain on *T. cruzi* C18:1-LPC. We carried out identical experiments with *T. cruzi* C18:0- and C18:2-LPC and found that both contained the acyl chain at the *sn*-1 position (data not shown).

To address the location of the double bonds in *T. cruzi* C18:1- and C18:2-LPC, we followed the protocols described by Hsu and Turk [69]. By comparing the MS^4^ spectra of Δ^9^- and Δ^6^-C18:1-LPC standards, we observed noticeably different fragmentation patterns of the acyl chain, especially in the relative abundance of fragment ions C_16_H_31_ (*m/z* 223), C_14_H_29_ (*m/z* 197), C_14_H_27_ (*m/z* 195), C_13_H_27_ (*m/z* 183), C_13_H_25_ (*m/z* 181), C_8_H_15_ (*m/z* 111), and C_8_H_13_ (*m/z* 109) (Fig. S3C). When *T. cruzi* C18:1-LPC (from ICA forms) was analyzed under the same MS conditions, the fragmentation pattern observed was consistent with a Δ^9^ double bond (Fig. S3C, bottom spectrum). The same type of experiment was conducted with *T. cruzi* C18:2-LPC (from ICA forms) and the resulting fragmentation was consistent with Δ^9,12^ double bonds (data not shown).

The POROS R1 protocol we have described here also enriched other LPC species, which included C22:6-LPC (*m/z* 574), C22:4-LPC (*m/z* 578), C16:0-LPC (*m/z* 502), and C16:1-LPC (*m/z* 500) that were also characterized by MS^n^ (Fig. S4). Most of these species, except for C22:6-LPC, had very low abundance and, in the case of Epi and Meta forms, could only be seen in the enriched 25%-*n*-propanol POROS R1 fraction. Even for TCT and ICA forms, MS^3^ and MS^4^ of the C16:1-, C22:4- and C18:0-LPC species could only be conducted with samples derived from the POROS R1 chromatography. This confirms that indeed this last fractionation step is necessary for the full characterization of low-abundance LPC species from complex phospholipid mixtures of *T. cruzi*. Using the current methodology, however, we were unable to detect any *bona fide* PAF species in any of the four parasite stages analyzed. Even employing the highly specific and sensitive MS approach, selective-ion monitoring (SIM), we could not detect any trace amounts of PAF species in *T. cruzi*. This was true not only for the POROS R1 25% n-propanol fractions, but for all fractions described in this study. Therefore, we surmise that if there were any PAF species in this parasite, the concentration levels would likely be below the detection limit of the MS approaches used in the present study.

**Figure 5:**
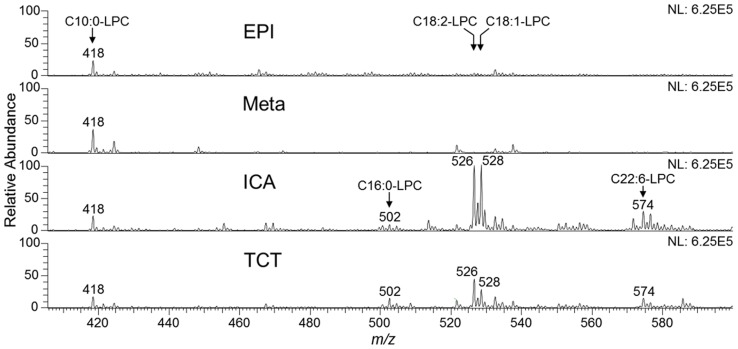
Quantification of LPC species in different life-cycle stages of *T. cruzi*. C10:0-LPC (*m/z* 418) was used as an internal standard for quantification of the most abundant *T. cruzi* LPC species. C10:0-LPC (18 nmoles) was added to all fresh parasite pellets prior to lipid extraction with C∶M (2∶1, v/v) and C∶M∶W (1∶2∶0.8, v/v/v), followed by Folch's partition. The Folch lower phase was analyzed by ESI-LIT-MS^n^. LPC species are indicated in the spectra. Epi, epimastigote; Meta, metacyclic trypomastigote; ICA, intracellular amastigote; TCT, tissue culture-derived trypomastigote.

After characterizing the different *T. cruzi* LPC species, we proceeded to quantify them using a synthetic C10:0-LPC as an internal standard. These analyses were conducted on freshly prepared parasite pellets to minimize the amount of time that *T. cruzi* phospholipases A1 and A2 [70] could have to act on PCs, leading therefore to an artificial increase in LPC levels. Overall, the lipid profiles of freshly prepared pellets were nearly identical to the profiles of previously frozen pellets of the same parasite forms (Figs. 2 and 5, and data not shown). The LPC quantification showed that the amount of C16:0-, C18:0-, C18:1- and C18:2-LPC present in the mammalian-dwelling forms of *T. cruzi* (ICA and TCT) were much higher than in the insect-dwelling (Epi, Meta) forms (Table 1). For instance, although the two most abundant LPC species found in all four parasite forms were C18:1- and C18:2-LPC, ICA forms had ∼44 and ∼35 times more of these species, respectively, than Epis. TCTs also showed very high levels of these lipids, having approx. 16–17 times more C18:1- and C18:2 LPC than Epis (Table 1). Interestingly, TCTs also contained the highest levels of C16:0-LPC, which has been shown to have immunosuppressant activities in the context of *T. cruzi* infection [17].

**Table 1:** The content of C18:2-, C18:1-, C18:0- and C16:0-LPC in different life-cycle stages of *T. cruzi*.

LPC species	Picomoles of LPC per 10^6^ cells	LPC amount (mol^−17^) per parasite^a^^, ^ b	Number of LPC molecules per parasite^c^	Parasite surface area (µm^2^)^d^	Number of LPC molecules per µm^2^ e	LPC ratio (in regard to Epi)^f^
**C18:2-LPC (** ***m/z*** ** 526.4)**						
Epi	1.4	0.15	0.90×10^6^	63	0.14×10^5^	1.0
Meta	0.6	0.05	0.32×10^6^	24	0.13×10^5^	0.4
ICA	**48.6**	5.10	**29.20×10^6^**	15	**19.47×10^5^**	**34.7**
TCT	**24.2**	2.55	**14.60×10^6^**	13	**11.23×10^5^**	**17.3**
**C18:1-LPC (** ***m/z*** ** 528.4)**						
Epi	1.1	0.11	0.66×10^6^	63	0.10×10^5^	1.0
Meta	0.4	0.05	0.26×10^6^	24	0.11×10^5^	0.4
ICA	**48.7**	5.00	**30.00×10^6^**	15	**20.00×10^5^**	**44.3**
TCT	**17.0**	1.75	**10.50×10^6^**	13	8.08×10^5^	**15.5**
**C18:0-LPC (** ***m/z*** ** 530.4)**						
Epi	0.4	0.04	0.24×10^6^	63	0.04×10^5^	1.0
Meta	0.7	0.07	0.40×10^6^	24	0.17×10^5^	1.8
ICA	**18.4**	1.72	**10.32×10^6^**	15	6.88×10^5^	**46.0**
TCT	4.9	0.50	2.70×10^6^	13	2.08×10^5^	**12.2**
**C16:0-LPC (** ***m/z*** ** 502.5)**						
Epi	**n/a** g	**n/a**	**n/a**	63	**n/a**	**n/a**
Meta	**n/a**	**n/a**	**n/a**	24	**n/a**	**n/a**
ICA	3.9	0.40	2.40×10^6^	15	1.60×10^5^	**n/a**
TCT	**7.9**	0.78	4.45×10^6^	13	3.42×10^5^	**n/a**

^*a*^The molar relative response factors (MRRF) of C10:0-LPC and LPC standards were used to calculate the amount of each LPC molecular species in Folch lower-phase fractions of *T. cruzi*.

^*b*^The number of parasites was determined before lipid extraction by counting live parasites in a hemocytometer. Values are means of three determinations. The standard deviation in all cases was <15%.

^*c*^Determined by multiplying the number of moles by the Avogadro's constant.

^*d*^Estimated based on the parasite's length and diameter as determined by scanning electron microscopy, and assuming that each parasite is cylindrical, as previous described [103].

^*e*^Obtained by dividing the number of LPC molecules per cell by the surface area of each parasite form.

^*f*^Obtained by dividing the amount of picomoles of LPC per 10^6^ cells of each parasite form (column 1) by the values obtained for Epi.

^*g*^Not analyzed due to absence or trace amounts of the compound.

To assess whether *T. cruzi* is also able to secrete LPC species to the extracellular milieu, we analyzed extracellular vesicles (EVs) and EV-free supernatant or conditioned medium (VF) of Epi and Meta forms, obtained as described [57]. Epi and Meta forms secrete two types of EVs, namely V2 and V16, which are vesicles obtained after 2 h and 16 h of ultracentrifugation (at 100,000×g), respectively. In both stages, V2 are larger vesicles resembling ectosomes (130–140 nm), whereas V16 are smaller vesicles resembling exosomes (70–90 nm). The final vesicle-free supernatant (VF) in both stages is virtually devoid of EVs [57]. As shown in Table S1 and Fig. S5, although Epis have higher amounts of C18:1- and C18:2-LPC than Metas in the total parasite pellet, the latter secrete much higher amounts of these phospholipids in the EV-free conditioned medium (mVF). Metas secrete 1.9 pmol of C18:1-LPC per 10^6^ cells in mVF and an additional 0.5 pmol associated with mV2 fraction. Similar values were found for C18:2-LPC in mVF and mV2 (Table S1). In comparison, Epis only secrete trace amounts of both LPCs in eV2. In our analyses, however, we were only able to detect trace amounts of C16:0-LPC in the V2, V16, and VF fractions of Epis and Metas (data not shown). Taken together, our data strongly indicate that C18:1-LPC, C18:2-LPC, and eventually other LPCs could actively be secreted by Metas during the early stages of the infection. Moreover, LPCs secreted by these parasites could contribute to the overall LPC pool in the plasma, saturating the lipid carrier proteins, and eventually activating PAF receptors. Most importantly, the relative concentration of C18:1-LPC could even be higher in the infected tissues, due to the continuous secretion of this phospholipid, associated or not with parasite EVs. Owing to the technical difficulty in obtaining enough parasites for preparation of EVs from TCTs or ICAs, we have not been able to conduct the same kind of quantification in these mammal-dwelling stages.

### Effect of LPC species on the aggregation of rabbit platelets

**Figure 6:**
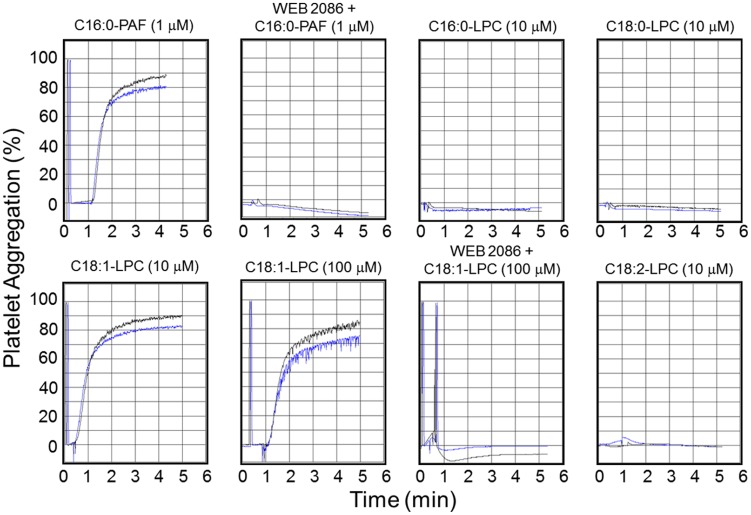
Activity of C16:0-PAF and different *T. cruzi* LPC species on the aggregation of rabbit platelets. Platelet aggregation assays were performed as described in Materials and Methods, using synthetic 16:0-PAF and C16:0-, C18:0-, C18:1-LPC, and purified C18:2-LPC. Control platelets or platelets pre-treated for 30 min with 10 µM WEB 2086 were assayed in the absence or presence of 1 µM C16:0-PAF or the LPC species at 10 µM (C16:0-LPC, C18:0-LPC, C18:1-LPC, and C18:2-LPC) and 100 µM (C18:1-LPC). Each lipid was tested in duplicate as indicated by black and blue curves in each graph.

Since we were unable to detect any PAF species in any of the four parasite stages analyzed, we hypothesized that the PAF-like molecule could be one or more of the lysophospholipids structurally characterized here. This assumption is also based on previous reports that certain LPC species seem to activate the PAFR [28], [33]. Moreover, purification of sufficient amounts of each *T. cruzi* LPC species for the bioassay (i.e., platelet aggregation) was not feasible. Therefore, we carried out the platelet aggregation assays using synthetic (C16:0-, C18:0-, C18:1-, and C22:6-LPC, at 1, 10, 100, and 1000 µM) or purified (C18:2-LPC) LPC species, and synthetic C16:0-PAF (at 1 µM; positive control). In a set of experiments platelets were pre-incubated for 30 min in the presence of 10 µM WEB 2086. As seen in Fig. 6, C16:0-LPC, C18:0-LPC, and C18:2-LPC failed to aggregate platelets at 10 µM, or at any other concentrations used (data not shown). Interestingly, C18:1-LPC was able to aggregate rabbit platelets at 10 and 100 µM (Fig. 6), but unlike PAF, failed to perform this activity at 1 µM (data not shown). WEB 2086 completely abolished platelet aggregation induced by C16:0-PAF and C18:1-LPC (at final concentrations of 1 µM and 100 µM, respectively) (Fig. 6). We were aware of the fact that depending upon the acyl chain length and degree of unsaturation, high concentrations (>30 µM) of LPCs could act as strong detergent and promote cell lysis [34]. Therefore, in our platelet aggregation assays, we used all LPCs at a maximal 10 µM concentration, except for C18:1-LPC, which was also tested at 100 µM. We did not observe platelet lysis up to 10 µM of any LPC tested, or even at 100 µM of C18:1-LPC.

### Comparative modeling of the PAF receptor

**Figure 7:**
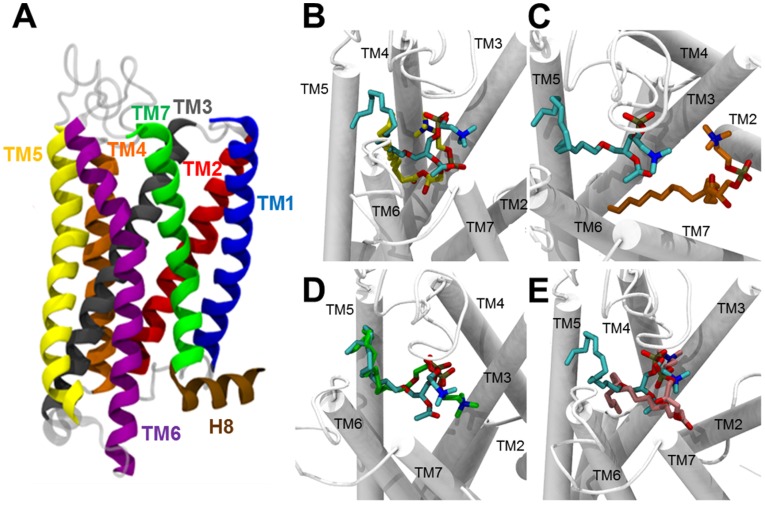
Structural representation of the PAFR model and its interaction with PAF and LPC species. (**A**) Side view of PAFR model. Each TM (alpha-helix) is indicated in a different color: TM1, blue; TM2, red; TM3, dark gray; TM4, orange; TM5, yellow; TM6, purple; TM7, green. The helix 8 (H8) at the end of C-terminus region is indicated (brown). (**B–E**) Comparison of binding modes of PAF and each LPC species to PAFR. (**B**) C16:0-PAF (cyan) and C16:0-LPC (yellow); (**C**) C16:0-PAF (cyan) and C18:0-LPC (orange); (**D**) C16:0-PAF (cyan) and C18:1-LPC (green); and (**E**) C16:0-PAF (cyan) and C18:2-LPC (pink). Transmembrane (TM) regions are represented as white rods.

To gain insights into the mechanism by which C18:1-LPC, but not C16:0, C18:0, C18:2, could induce platelet aggregation mediated by PAFR, we decided to build a 3D-structural model of the PAFR, since a high-quality model was not available in the literature. The structural features of our PAFR model are shown in Figure 7A. As a member of the class A of GPCR superfamily, the PAFR model encompasses an extracellular N-terminus, followed by seven transmembrane (TM) α-helices (TM1, TM2, TM3, TM4, TM5, TM6, and TM7) (Fig. S6A), connected by three extracellular loops (EL1, EL2, and EL3) (Fig. S6B) and three intracellular cytoplasmic loops (CL1, CL2, and CL3) (Fig. S6C) and, finally, a short α-helix (H8) at the intracellular C-terminus (Fig. 7A). Additionally, the spatial arrangement of the 7-TM bundle constitutes a hydrophilic cavity covered by EL2, which is described as a ligand-binding pocket (Figs. 7A and S6B) [68], [71]–[76].

### Structural validation of the PAFR model

The I-TASSER methodology is very accurate for the construction of protein models when the sequence identity between the target sequence and the template protein drops below 30%, where lack of a high-quality structure match may provide substantial alignment errors and, consequently, poor quality models. In addition, the I-TASSER methodology has already been used for modeling other GPCRs [68], [76]. The structural validation of the PAFR model was performed using three programs: PROCHECK [65], ERRAT [66], and PROQM [67]. The stereochemical quality of the PAFR model was evaluated using PROCHECK program. According to an analysis of 118 known protein structures, a good quality model would be expected to have over 90% in the most favored regions. The analysis of the first Ramachandran plot revealed that 97.8% of the amino acid residues are located in the most favorable (92.0%) and additional allowed (5.8%) regions, confirming the excellent quality of the PAFR model described here (Fig. S7). The analysis of the second Ramachandran plot, which considers only Φ and ψ angles for Gly residues, showed that all 11 Gly residues in the primary sequence of PAFR were in allowed regions, with favorable combinations of angles Φ and Ψ (Fig. S8). The properties of the main and side chains for PAFR model were also evaluated by PROCHECK program, which showed acceptable values for our model when compared to experimentally-determined protein structures (Fig. S9 and S10). The analysis of distortions in the geometry of the PAFR model residues was also analyzed using PROCHECK (Fig. S11). The parameters analyzed were the bond lengths and angles, including atoms of the main and side chains, and the results were considered acceptable for our PAFR model. In addition, we used the ERRAT program for verifying errors in non-bonded atom-atom interactions in our model. The error values are plotted as a function of the position of a sliding residue in the window [66]. According to this analysis, structures at high resolutions, generally produce values around 95% or higher. However, for protein structures at lower resolutions (2.5 to 3 Å), the average overall quality factor can be around 91%. The ERRAT analysis gave an overall quality factor value of 87% for our PAFR model (Fig. S12). Although low for crystal structures, this value is acceptable for protein models [67]. Finally, we analyzed the quality of the three-dimensional structure of our model at local and global levels, using PROQM program [66] (Fig. S13). This program uses a score function to evaluate structures of membrane proteins, including GPCRs [67], [77], [78]. Thus, to each residue of the protein model is given a score, producing, consequently, an overall quality factor. The value of the overall quality factor generated by server PROQM ranges from 0 to 1, indicating a model of poor and excellent quality, respectively [64]. The PROQM analysis gave a global quality score of 0.717 for our PAFR model (Fig. S13), which is similar to the score values for crystallographic structures of GPCR [67].

### Molecular docking

**Figure 8:**
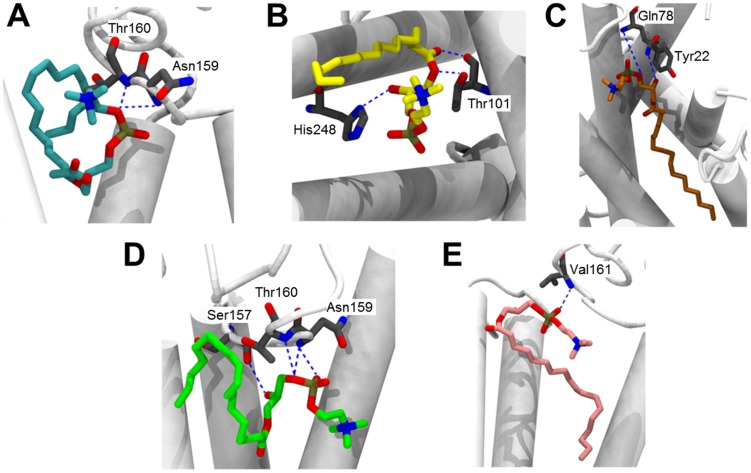
Hydrogen bonds between different lysophospholipid ligands and PAFR. Hydrogen bonds are represented by blue interrupted lines. (**A**) C16:0-PAF; (**B**) C16:0-LPC; (**C**) C18:0-LPC; (**D**) C18:1-LPC; and (**E**) C18:2-LPC. Nitrogen atoms are shown in blue, oxygen in red, and ligand carbon chains are filled with the specific color for each ligand: C16:0-PAF, cyan; C16:0-LPC, yellow; C18:0-LPC, orange; C18:1-LPC, green; C18:2-LPC, pink.

To compare the binding mode of all ligands (C16:0-PAF, and C16:0-, C18:0-, C18:1-, and C18:2-LPC) to the PAFR model, we carried out ten docking simulations for each ligand (totalizing 50 poses per ligand) and, consequently, the poses with the lowest energy for each ligand was selected for analysis. The comparison between C16:0-PAF and each LPC species is represented in Fig. 7B–E and Fig. S14. The molecular docking study showed that C18:1-LPC and C16:0-PAF have similar modes of interaction with PAFR (Fig. 7D and Fig. S14C). On the other hand, no other LPC species was able to interact with PAFR in a similar way, which corroborates the platelet aggregation assays (Fig. 6). Since PAF and LPC have different structures, RMSD matrix was not calculated. Instead, distances between the heteroatoms of the functional groups (amino, phosphate, and acyl groups) of C16:0-PAF and all LPC species were measured. The nitrogen of the amino group, the phosphorous of the phosphate group, and the oxygen linking the glycerol backbone (at *sn*-1) to the carbonyl group of the acyl chain were chosen and all distances between the ligands are presented in Table 2. The distances between heteroatoms of C16:0-PAF and C18:1-LPC are clearly lower than the distances between C16:0-PAF and other LPC species (C16:0, C18:0, and C18:2), with the exception of the amino group of C18:2-LPC and C16:0-PAF, confirming the similarities between C16:0-PAF and C18:1-LPC binding modes. The energy of the interactions between PAFR and each ligand was also analyzed (Table 3). The hydrogen bonds and repulsive steric interactions were mapped using a ligand-map algorithm, generated by the MVD program [68]. The hydrogen bonds are represented in Figure 8. C16:0-PAF and C18:1-LPC interact with the same amino acids (i.e., Asn159 and Thr160) of the PAFR model. Other LPCs do not present the same pattern of interaction. Interestingly, C18:1-LPC is able to additionally interact with Ser157, through its hydroxyl group, in a very strong way (Fig. 8 and Table 3). Although C16:0-, C18:0-, and C18:2-LPC also form hydrogen bonds with amino acids, these interactions occur in a different region of the PAFR model and with lower strength. Finally, the amino group either of C16:0-PAF or any of the LPC species did not seem to interact to our PAFR model (Fig. S15).

**Table 2:** Distances between the nitrogen, phosphorous, and oxygen atoms in functional groups of C16:0-PAF and each LPC species.

Ligands	Atom^a^		
	Nitrogen (Å)	Phosphorous (Å)	Oxygen (Å)
PAF vs. C16:0-LPC	19.0	13.6	5.6
PAF vs. C18:0-LPC	6.1	11.1	12.0
PAF vs. C18:1-LPC	6.1	1.2	1.7
PAF vs. C18:2-LPC	5.5	3.5	9.6

^*a*^This refers to the nitrogen of the amino group, the phosphorous of the phosphate group, and the oxygen at *sn*-1, linking the glycerol backbone to the carbonyl group of the acyl chain.

**Table 3:** Summary of the interactions of each ligand with PAFR model.

Ligand	H-bond energy (Kcal.mol^−1^)	Residues (H-bond interaction)	Steric interaction energy by PLP^a^ (Kcal.mol-^1^)	Residues (steric interactions)	MolDock score (Kcal.mol^−1^)
C16:0-PAF	−2.84194	Asn159, Thr160	−11.453	Asn159, Thr160, Pro183, His248, Trp255, Thr256, Glu259	−167.335
C16:0-LPC	−1.33205	Thr101, His248	−18.271	Phe97, Phe98, Thr101, Tyr102, Ile187, Ile191, His248, Gln252, Trp257	−161.28
C18:0-LPC	−3.43936	Tyr22, Gln78	−0.066	Tyr22, Phe66, Ile74, Gln78, Trp83, Phe97, Val105, Phe241, Leu279, Leu282, Ser283	−161.634
C18:1-LPC	−7.24856	Ser157, Asn159, Thr160	−3.676	Phe98, Ser157, Asn159, Thr160, Ile187, Gln252, Trp255, Thr256, Glu259	−174.597
C18:2-LPC	−2.32134	Val161	−21.470	Phe98, Tyr102, Thr158, Thr160, Val161, Ile191, Trp255, Gln276, Leu279	−169.19

a Piecewise linear potential.

## Discussion

Previous results from our group showed that *T. cruzi* synthesizes a PAF-like phospholipid capable of aggregating rabbit platelets [14]. Aiming at the purification and structural characterization of this putative PAF-like molecule, we developed a fractionation protocol that proved efficient for the enrichment of PAF and other lysophospholipids such as LPAF and LPC. Tandem MS and bioactivity data obtained in the present study revealed that the *T. cruzi* PAF-like phospholipid is in fact an LPC, namely *sn*-1 C18:1(Δ^9^)-LPC. However, we do not discard the possibility that very low amounts of a *bona fide* PAF species might still be synthesized by this parasite. The MS^n^ assignments for the C18:1-LPC we provide here are in agreement with previous reports [54], [56], but somewhat different from the results obtained by Smith *et al.* [41]. Specifically, these authors analyzed a lipid species with a nearly identical fragmentation pattern as what we have identified here as C18:1-LPC, but annotated it as a novel C16:1-alkenyl-PAF. In their study, an ion species at *m/z* 265 was characterized as being derived from the neutral loss of acetyl and methyl groups from the fragment ion at *m/z* 339. In addition, a fragment ion at *m/z* 247 was proposed to be derived from the loss of water from the ion at *m/z* 265. Conversely, we show here that the non-lithiated [R_1_CO^+^]^+^ and [R_1_CO^+^ - H_2_O]^+^ fragment ions at *m*/z 265 and 247, respectively, are in fact derived from a C18:1-acyl chain at the *sn*-1 position. This is further confirmed by the presence of lithiated ([R_1_CO_2_H+Li]^+^) fragment ion from oleyl chain at *m*/z 289. Our assignments of C18:1-LPC are in complete agreement with those reported by Hsu et al. [54]. Smith et al. [41] also proposed the ion species at *m/z* 223 as being the protonated C16:1-alkenyl chain from the *sn*-1 position of that putative PAF species. We also observed the same fragment ion in both synthetic and *T. cruzi*-derived C18:1-LPC, but we assigned it as derived from the fragmentation of the acyl chain. Taking all these facts into consideration, it is likely that Smith et al. [41] have mistakenly identified C18:1-LPC as C16:1-PAF.

Here we show that *T. cruzi* synthesizes at least five species of LPC and that only *sn*-1 C18:1(Δ^9^)-LPC was able to promote rabbit platelet aggregation. These LPCs could be generated by the action of host- and/or parasite-derived phospholipase(s) A1 and/or A2 on diacyl-PCs. A PLA1 has already been well characterized in *T. cruzi* trypomastigote and amastigote forms (RA, Cvd, and K98 strains) [79], [80] and *T. brucei* [81]. However, since we could only identify *sn*-1 C18:0-, C18:1-, and C18:2-LPC, we believe that the parasite PLA1 is not playing a major role in the generation of these LPCs, at least under the experimental conditions used in this study. Nevertheless, we could not discard the possibility that the *T. cruzi* PLA1 might be important for the generation of LPC species using different experimental conditions and/or other parasite strains, as previously described [79], [80]. Therefore, most likely the LPC species identified in this study were generated by the action of a PLA2 from the host and/or parasite. No PLA2 has so far been purified and fully characterized in *T. cruzi*, despite the fact that at least four putative PLA2 genes have been annotated in the parasite genome (TriTrypDB TcCLB.510743.50, TcCLB.510659.257, TCSYLVIO_005843, and Tc_MARK_4470) [70]. Although a PLA2-like activity has been previously reported in epimastigotes [82], no further characterization of this enzymatic activity has been published. Moreover, using more rigorous enzymatic assay conditions, Belauzaran et al. [70] were unable to detect any PLA2 activity in *T. cruzi* epimastigote supernatants. This raises the possibility that the *T. cruzi* LPCs identified here could have been generated by the action of a host-derived PLA2 and/or by a parasite-derived enzyme that is expressed in much higher levels in the mammal-dwelling stages. Further studies are needed to address this important point.

A direct correlation between increased platelet reactivity and the incidence of acute coronary diseases has been described [83]. Accordingly, there is an increase in platelet aggregation associated with ischemia, myonecrosis, and myocarditis in both acute and chronic stages of Chagas disease [2], [4], [5], [84]. Noteworthy, *T. cruzi* also synthesizes TXA2, which modulates several pathophysiological aspects of Chagasic cardiomyopathy [12]. It is not surprising that *T. cruzi* produces at least two platelet activators, given the importance of platelet aggregation in the progression of Chagas disease. Our bioactivity data with C18:1-LPC are in agreement with other results in the present study, namely the platelet aggregation assays and molecular docking predictions. We had also previously demonstrated that the putative PAF-like phospholipid was able to trigger the differentiation of *T. cruzi* epimastigotes into metacyclic trypomastigotes *in vitro* [14]. This effect was abolished by WEB 2086, a classic competitive PAF antagonist that binds specifically to PAF receptors [85]. The latter result along with labeling *T. cruzi* epimastigotes with polyclonal antibody raised against mouse PAFR, in immunofluorescence assays, strongly indicated that *T. cruzi* might have putative PAFR both at the cell surface and intracellularly [14]. In fact, WEB 2086 inhibited all PAF effects upon trypanosomatids described to date [86]–[88].

LPC has messenger functions, binding to a specific receptor and not acting through physicochemical effects on the plasma membrane of the target cell [27]. Several receptors for LPC, such as G2A, GPR4, and IP, and receptors for TXA2 (TP) and PAF (PAFR), have been described [24]–[32]. In the present study, we show that the platelet aggregation promoted by C18:1-LPC was abrogated by WEB 2086. This result is highly suggestive that *T. cruzi*-C18:1-LPC may be able to trigger platelet stimulation through a ligand-receptor system. Accordingly, LPC is known to induce intracellular signals [89] and mediate cytokine secretion [90], both through PAFR. Interestingly, the *R. prolixus*-derived C16:0-LPC is able to prevent platelet aggregation triggered by PAF [17], [19]. We may hypothesize that C16:0-LPC acts as a PAF antagonist, binding to its receptor. Indeed, here we show molefcular docking calculations, which are suggestive that C16:0-, C18:0-, and C18:2-LPC could be lodged within the ligand-binding site of PAFR, preventing PAF actions. On the other hand, the predicted binding pose for C18:1-LPC evokes the possibility that this molecule and C16:0-PAF exhibit similar modes of interaction with PAFR. These results can be partially explained by the fact that, depending on the length and degree of unsaturation of the acyl-chain, each LPC species triggers different cellular activities [30], [34], [35]. Apparently, each of these LPC species binds to different receptors, probably because of the tridimensional structure of these phospholipids. For instance, TP receptors are involved in the attenuation of vascular relaxation mediated by C18:2- and C20:4-LPC, but not C16:0- or C18:1-LPC [30]. Additionally, similar to C16:0-PAF, C18:1-LPC exhibited the ability to elicit a rapid, receptor-mediated oxidative burst, through generation of reactive oxygen species (ROS) in neutrophils. On the other hand, C16:0- and C18:0-LPC did not share the same activity [34]. Consistently, our docking calculations show that both C16:0-PAF and C18:1-LPC structures interact with PAFR model with Asn169 and Thr170 residues via hydrogen bonds. These amino acid residues are localized at the extracellular loop EL2, which is involved in the ligand recognition and receptor activation in the superfamily of the GPCRs [91]. The number and the chain length of the fatty acids of lipoproteins are also substantially important for the induction of signaling through Toll-like receptor 2 (TLR2) [92]. A cross-talk between TLR and GPCR has been described, which is essential for cellular signaling in the absence of TLR natural ligands. This cross-talk is basically a molecular organizational GPCR signaling platform that promotes the transactivation of TLR, through potentiation of Neuraminidase 1 and matrix metalloproteinase-9 at the cell surface [93]. Intriguingly, LPC derived from the human pathogen *Schistosoma mansoni* activates TLR-2-dependent signaling involved in eosinophil activation and recruitment, probably through cross-talk to a GPCR [94]. More recently, we have shown that various species of LPC containing different acyl chain lengths and degrees of unsaturation may induce proinflammatory response mediated by TLR4- or TLR2/TLR1-dependent signaling pathway in HEK293A cells [95]. Interestingly, a mixture of LPC species (mainly C16:0- and C18:0-LPC) could counteract TLR4-mediated signaling pathway triggered by *E. coli* O111:B4 lipopolysaccharide (LPS), suggesting therefore a dual role of this lysophospholipid in immunoregulation.

Over time, LPC was found not to be exclusively of mammalian origin and was described in several other organisms [96]. Trypanosomatids tend to be rich in PC and LPC [36]–[39], [97]. However, in most of these cases, the presence of LPC has only been inferred indirectly and there still remains a general lack of information in the literature regarding the chemical structure and function of the LPC species in most organisms other than mammals. The results described in the present study show that *T. cruzi* is able to generate different LPC species and suggest that the levels of individual LPC species are tightly regulated in the course of this parasite cell cycle. In fact, the LPC levels are notably high in the mammal-dwelling infectious trypomastigote and intracellular amastigote stages. Taken together with the fact that there was no exogenous source (e.g., fetal bovine serum) of lipids in the TAU culture medium used for the differentiation of epimastigotes into metacyclic trypomastigotes, our data indicate that LPC species might be generated endogenously by this organism.

The saliva of *R. prolixus* is a source of LPC, which may act as an enhancing factor for Chagas disease [17], [18]. LPC, TXA2, and PAF are lipid mediators that share a common chemical structure, biosynthetic pathways [98] and the ability to activate platelets [99]–[101]. Previous results from our group have shown that PAF-treated *T. cruzi* is far more infective towards both mouse macrophages [14] and the insect vector *R. prolixus* [102]. TXA2 synthesized by *T. cruzi* controls the proliferation of the parasite and resulting inflammatory response to infection in the mouse [12]. One may then speculate that host-vector-parasite co-evolutionary relationships may be involved in the maintenance or change in the enzymatic pathways for the synthesis and degradation of these molecules, which ultimately may dictate the success of *T. cruzi* infection.

In conclusion, here we demonstrate that *T. cruzi* synthesizes at least five LPC species, C16:0-, C18:0, C18:1-, C18:2-, and C22:6-LPC. The most abundant species are C18:2- and C18:1-LPC, being the latter the only one able to aggregate rabbit platelets, probably through a PAFR. This result was supported by molecular docking study of the interactions between LPC species and a PAFR model. These analyses showed that C18:1-LPC was able to interact with the PAFR model in a fashion similar to PAF. More studies on LPC metabolism in *T. cruzi* and relationship of the parasite with the mammalian and invertebrate hosts could, therefore, lead to the discovery of putative targets for novel therapies and control for Chagas disease.

## Supporting Information

Figure S1 Fractionation of phospholipid standards using POROS R1 perfusion chromatography. A mixture of phospholipid standards, containing synthetic C16:0-*lyso*-PAF (LPAF) (*m/z* 488.4), C16:0-*lyso*-LPC (LPC) (*m/z* 502.4), C16:0-PAF (PAF) (*m/z* 530.5), and purified diacyl-PCs (700–900 *m/z* range), was suspended in HPLC-grade water and applied onto the POROS R1 mini-column, which was eluted with a 0%–50% *n*-propanol gradient. All fractions were diluted in methanol containing 5 mM LiOH and analyzed by direct infusion in an LTQXL ESI-LIT-MS, in positive-ion mode.
(TIF)

Figure S2 ESI-LIT-MS^n^ analysis of major *T. cruzi* ion species enriched by POROS R1 fractionation. (**A**) ESI-LIT-MS^2^ spectra. Phospholipid standards (C16:0-PAF, C18:0-LPC, and C18:1-LPC) or purified *T. cruzi* phospholipids from the POROS R1 25% *n*-propanol fractions were diluted in methanol containing 5 mM LiOH and then infused directly into the LTQXL MS using an Advion Triversa NanoMate nanoelectrospray system. Major parent-ion species observed in the MS spectra (Fig. 2) were subjected to MS^2^ fragmentation. (**B**) ESI-LIT-MS^3^ spectra of selected ion species found in (**A**). Ion species corresponding to a neutral loss of 59 *m/z* (trimethylamine) from the parent ion were selected for MS^3^ fragmentation.
(TIF)

Figure S3 Determination of the acyl chain and double bond positions on *T. cruzi* C18:1-LPC. (**A–B**) Acyl chain position analysis. The *sn*-1 C18:1-LPC and *sn*-2 C18:1-LPC regioisomer standards were generated by treatment of 18:1(Δ^9^-*cis*)-diacyl-PC with PLA2 and PLA1, respectively, as described in Materials and Methods. (**A**) MS^2^ analysis. All LPCs were diluted in methanol containing 2.5 mM NaCl and analyzed by direct infusion using an Advion Triversa NanoMate nanoelectrospray system coupled to an LTQXL MS. MS^2^ spectra were acquired in positive-ion mode. (**B**) MS^3^ analysis. All LPCs were diluted in methanol containing 2.5 mM LiOH and analyzed by MS^3^ (MS^2^ 528.7→MS^3^ 469.3), under the same MS experimental conditions. (**C**) Double-bond position analysis. The *sn*-1 C18:1(Δ^6^-*cis*)-LPC and *sn*-1 C18:1(Δ^9^-*cis*)-LPC standards were generated by treatment of 18:1(Δ^6^-*cis*)-diacyl-PC and 18:1(Δ^9^-*Cis*)-diacyl-PC with PLA2, respectively, as described in Materials and Methods. The position of the unsaturation on the acyl moiety of *T. cruzi* C18:1-LPC was determined by comparing the MS^4^ fragmentation pattern of this ion (MS^2^ 528.7→MS^3^ 469.3→MS^4^ 339.3) to the fragmentation pattern of *sn*-1 C18:1(Δ^6^-*cis*)-LPC and *sn*-1 C18:1(Δ^9^-*cis*)-LPC under identical conditions to (**B**). Diagnostic ions (bold) are indicated.
(TIF)

Figure S4 Tandem ESI-LIT-MS spectra of the low-abundance *T. cruzi* LPC-species enriched by POROS R1 fractionation. *T. cruzi* phospholipids from the 25% *n*-propanol fractions were diluted in methanol containing 5 mM LiOH and then infused directly into an LTQXL ESI-LIT-MS. Selected peaks were sequentially fragmented (MS^2^-MS^4^) by CID, as follows: C16:0-LPC (MS^2^ 502→MS^3^ 443→MS^4^ 313); C16:1-LPC (MS^2^ 500→MS^3^ 441→MS^4^ 311); C22:4-LPC (MS^2^ 578→MS^3^ 519→MS^4^ 389); and C22:6-LPC (MS^2^ 574→MS^3^ 515→MS^4^ 385).
(TIF)

Figure S5 Quantification of LPC species in extracellular vesicles (EV) and EV-free supernatant of *T. cruzi*. C10:0-LPC (*m/z* 418) was used as an internal standard for quantification of the most abundant *T. cruzi* LPC species. C10:0-LPC was added to fresh pellets or EV preparations from Epis and Metas, prior to lipid extraction with C∶M (2∶1, v/v) and C∶M∶W (1∶2∶0.8, v/v/v), followed by Folch's partition. The Folch lower phase was analyzed by ESI-LIT-MS. C18:1- and C18:2-LPC are indicated at *m/z* 528 and 526, respectively. ePellet, Epi total pellet; eV2, Epi-derived ectosomes; eV16, Epi-derived exosomes; eVF, Epi-derived EV-free supernatant (or conditioned medium); mPellet, Meta total pellet; mV2, Meta-derived ectosomes; mV16, Meta-derived exosomes; mVF, Meta-derived EV-free supernatant.
(TIF)

Figure S6 Structural representation of the PAFR model. (**A**) Arrangement of the 7 transmembrane (TM) helices forming a cavity. (**B**) The extracellular loops (EL1-EL3). (**C**) The intracellular loops (CL1-CL3). Each TM (alpha-helix) is indicated in a different color. TM1, blue; TM2, red; TM3, dark gray; TM4, orange; TM5, yellow; TM6, purple; TM7, green.
(TIF)

Figure S7 Main Ramachandran plot of the PAFR model. The main Ramachandran plot represents all amino acid residues by squares, whereas the glycine (Gly) residues are separately identified by black triangles, because these are not restricted to the regions in the generic Ramachandran plot. Red (A, B, and L) and dark yellow (a, b, l, and p) colors correspond to combinations of phi (Φ) and psi (Ψ) torsion angles of amino acid residues in the most favorable and additional allowed regions, respectively. In addition, pale yellow (∼a, ∼b, ∼l, and ∼p) and white colors represent less favorable and disallowed regions, respectively. The amino acid residues lying in those regions are highlighted in red.
(TIF)

Figure S8 Ramachandran plot for Gly residues of the PAFR model. The second Ramachandran plot considers only Φ and ψ angles for Gly residues, where the number in parenthesis indicates the total number of these residues in the primary sequence of PAFR. The favorable combinations of these angles are represented by green areas and the values of standard deviations greater than 2.5 Å are labeled in red in the graph, describing combinations of disallowed angles. All Gly residues are in allowed regions, with favorable combinations of angles Φ and Ψ.
(TIF)

Figure S9 Analysis of the main-chain parameters of the PAFR model. The six graphs on the main chain parameters plot show how the PAFR model (represented by the black square) compares with a database of well-refined protein structures. The blue band in each graph represents the results from the well-refined protein structures; the central line is the least squares fitting to the mean trend as a function of resolution, whereas the width of the band on either side of it corresponds to a variation of one standard deviation from the mean. The six properties plotted are: (a) Ramachandran plot quality assessment; (b) peptide bond planarity; (c) number of bad contacts per 100 residues; (d) α-carbon tetrahedral distortion (this property is measured by calculating the standard deviation of the zeta torsion angle); (e) main chain hydrogen bond energy; and (f) overall G-factor (the overall value is obtained from an average of all the different G-factors for each residue in the protein structure). The G-factor provides a measure of how “normal”, or alternatively how “unusual”, a given stereochemical property is and, in PROCHECK, it is computed for the following properties: Φ-Ψ combination, chi1-chi2 (X_1_-X_2_; side chain torsion angles) combination, X_1_ torsion for those residues that do not have a X_2_, combined X_3_ and X_4_ torsion angles, omega torsion angles, main chain bond lengths, and main chain bond angles.
(TIF)

Figure S10 Analysis of the side-chain parameters of the PAFR model. In a similar way to the main-chain analysis, the side-chain analysis shows how the PAFR model (black square) compares with well-refined protein structures. This analysis evaluates five properties: (a) standard deviation values of the Chi-1 *gauche* minus torsion angles; (b) standard deviation values of the Chi-1 *trans*; (c) standard deviation values of the Chi-1 *gauche* plus; (d) pooled standard deviation of Chi-1 torsion angles; and (e) standard deviation values of the Chi-2 *trans* torsion angles.
(TIF)

Figure S11 Analysis of geometrical distortions of the PAFR model. The parameters analyzed were: lengths and bond angles, including atoms of the main and side chains. This analysis shows the amino acid residues with distorted geometry, including their ideal values (in blue), those found in the model (in red), and the difference between these values (in green).
(TIF)

Figure S12 ERRAT analysis of the PAFR model. Errors in non-bonded atom-atom interactions of the PAFR model were verified by this analysis. The error values were plotted as a function of the position of a sliding residue in the window. An overall quality factor value of 87% was observed, thus validating the PAFR model.
(TIF)

Figure S13 ProQM analysis of the PAFR model. A score was given to each residue of the protein model, which lead to a global quality factor of 0.717, which corroborates the other modelling analyses.
(TIF)

Figure S14 Comparison of binding modes of PAF and each LPC species to PAFR. (A) C16:0-PAF (cyan) and C16:0-LPC (yellow); (B) C16:0-PAF (cyan) and C18:0-LPC (orange); (C) C16:0-PAF (cyan) and C18:1-LPC (green); (D) C16:0-PAF (cyan), and C18:2-LPC (gray). Transmembrane (TM) regions are represented as white rods.
(TIF)

Figure S15 Unfavorable steric interactions of PAF and LPCs with PAFR. Heteroatoms are represented by different colors in structures: nitrogen atoms are shown in blue, oxygen in red, and carbon atoms of amino acids in gray. In ligands, carbon chains are represented by different colors: (A) C16:0-PAF, cyan; (B) C16:0-LPC, yellow; (C) C18:0-LPC, orange; (D) C18:1-LPC, green; and (E) C18:2-LPC, pink.
(TIF)

Table S1 Quantification of LPC species in extracellular vesicles (EVs) and EV-free supernatant of epimastigotes and metacyclic trypomastigote forms.
(DOCX)
