# Supplementary figures and images for: Structural and Functional Analysis of a Platelet-Activating Lysophosphatidylcholine of Trypanosoma cruzi
Source: PLoS Negl Trop Dis. 2014 Aug 7;8(8):e3077. doi: 10.1371/journal.pntd.0003077 (PMC4125143; doi:10.1371/journal.pntd.0003077)

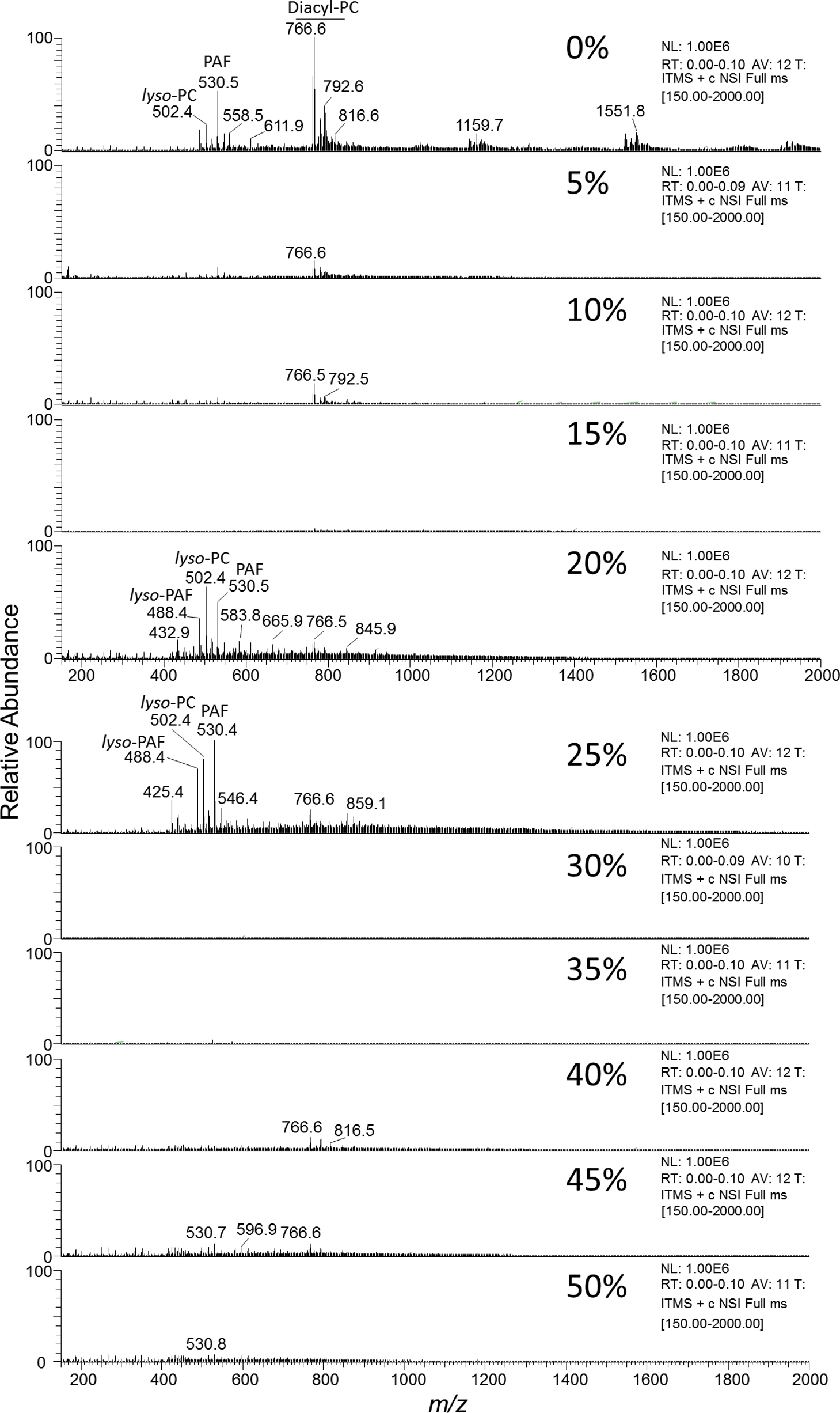

Supplement: Figure S1 — Fractionation of phospholipid standards using POROS R1 perfusion chromatography. A mixture of phospholipid standards, containing synthetic C16:0-lyso-PAF (LPAF) (m/z 488.4), C16:0-lyso-LPC (LPC) (m/z 502.4), C16:0-PAF (PAF) (m/z 530.5), and purified diacyl-PCs (700–900 m/z range), was suspended in HPLC-grade water and applied onto the POROS R1 mini-column, which was eluted with a 0%–50% n-propanol gradient. All fractions were diluted in methanol containing 5 mM LiOH and analyzed by direct infusion in an LTQXL ESI-LIT-MS, in positive-ion mode. (TIF) [file pntd.0003077.s001.tif]

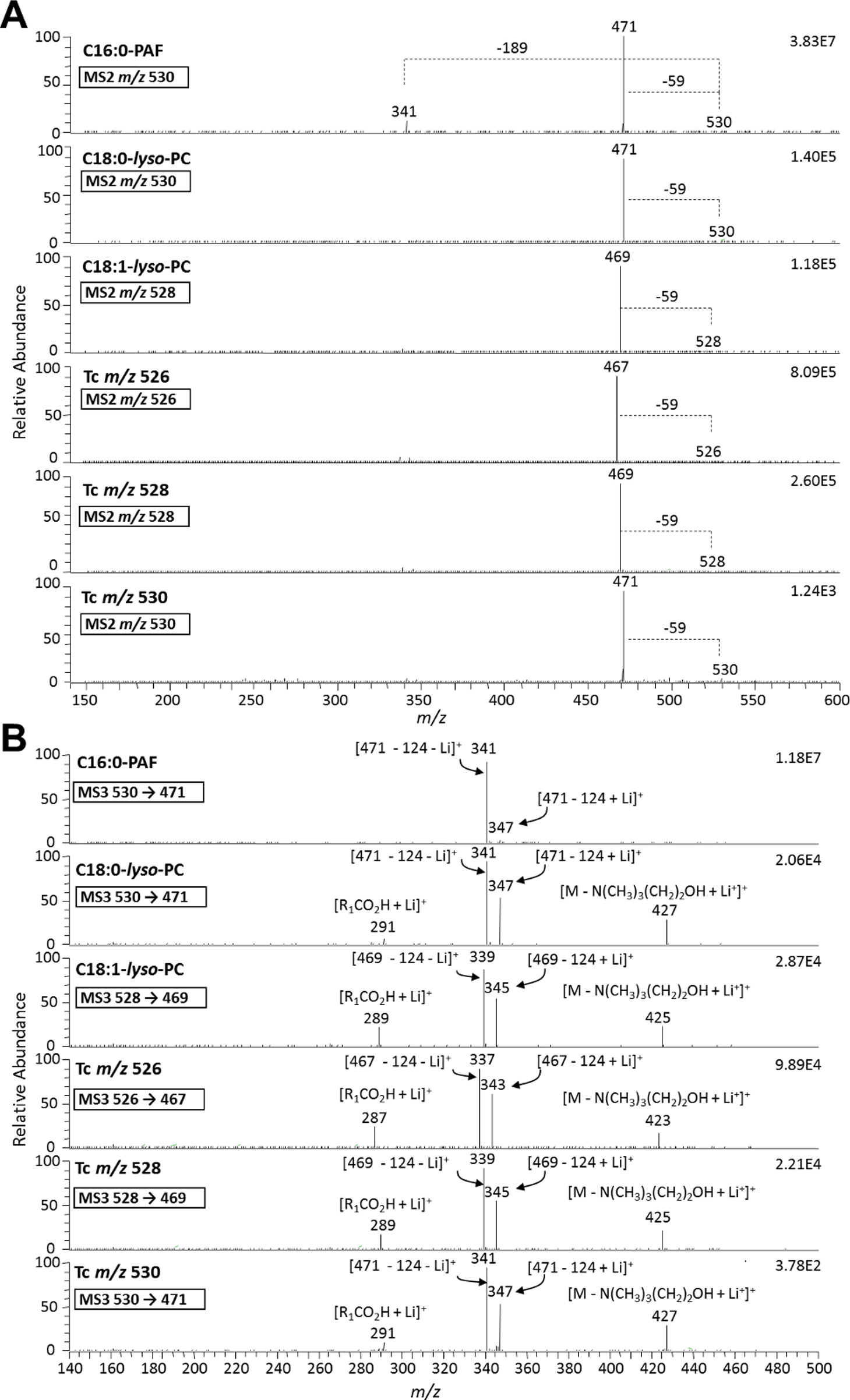

Supplement: Figure S2 — ESI-LIT-MSn analysis of major T. cruzi ion species enriched by POROS R1 fractionation. (A) ESI-LIT-MS2 spectra. Phospholipid standards (C16:0-PAF, C18:0-LPC, and C18:1-LPC) or purified T. cruzi phospholipids from the POROS R1 25% n-propanol fractions were diluted in methanol containing 5 mM LiOH and then infused directly into the LTQXL MS using an Advion Triversa NanoMate nanoelectrospray system. Major parent-ion species observed in the MS spectra (Fig. 2) were subjected to MS2 fragmentation. (B) ESI-LIT-MS3 spectra of selected ion species found in (A). Ion species corresponding to a neutral loss of 59 m/z (trimethylamine) from the parent ion were selected for MS3 fragmentation. (TIF) [file pntd.0003077.s002.tif]

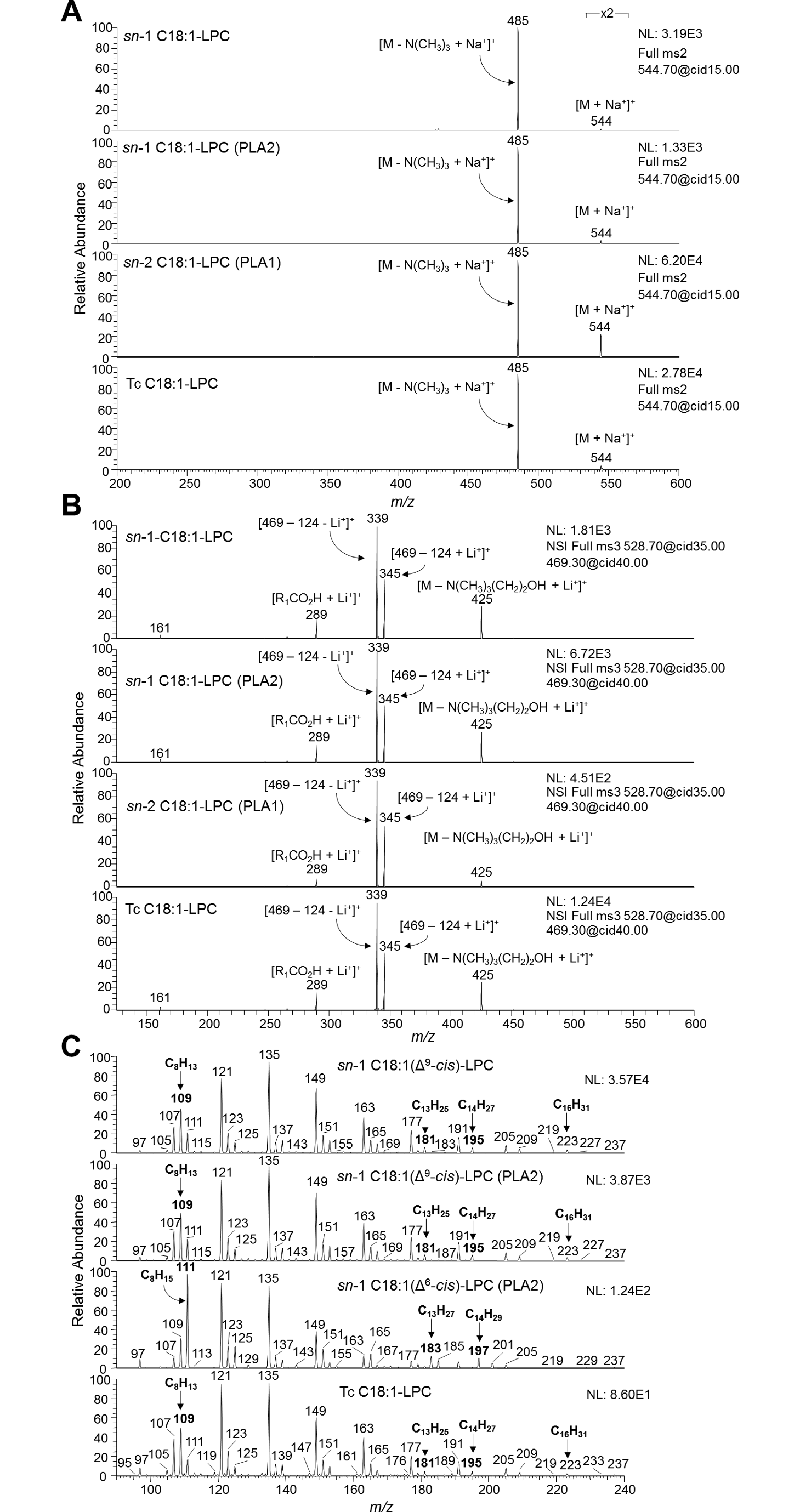

Supplement: Figure S3 — Determination of the acyl chain and double bond positions on T. cruzi C18:1-LPC. (A–B) Acyl chain position analysis. The sn-1 C18:1-LPC and sn-2 C18:1-LPC regioisomer standards were generated by treatment of 18:1(Δ9-cis)-diacyl-PC with PLA2 and PLA1, respectively, as described in Materials and Methods. (A) MS2 analysis. All LPCs were diluted in methanol containing 2.5 mM NaCl and analyzed by direct infusion using an Advion Triversa NanoMate nanoelectrospray system coupled to an LTQXL MS. MS2 spectra were acquired in positive-ion mode. (B) MS3 analysis. All LPCs were diluted in methanol containing 2.5 mM LiOH and analyzed by MS3 (MS2 528.7→MS3 469.3), under the same MS experimental conditions. (C) Double-bond position analysis. The sn-1 C18:1(Δ6-cis)-LPC and sn-1 C18:1(Δ9-cis)-LPC standards were generated by treatment of 18:1(Δ6-cis)-diacyl-PC and 18:1(Δ9-Cis)-diacyl-PC with PLA2, respectively, as described in Materials and Methods. The position of the unsaturation on the acyl moiety of T. cruzi C18:1-LPC was determined by comparing the MS4 fragmentation pattern of this ion (MS2 528.7→MS3 469.3→MS4 339.3) to the fragmentation pattern of sn-1 C18:1(Δ6-cis)-LPC and sn-1 C18:1(Δ9-cis)-LPC under identical conditions to (B). Diagnostic ions (bold) are indicated. (TIF) [file pntd.0003077.s003.tif]

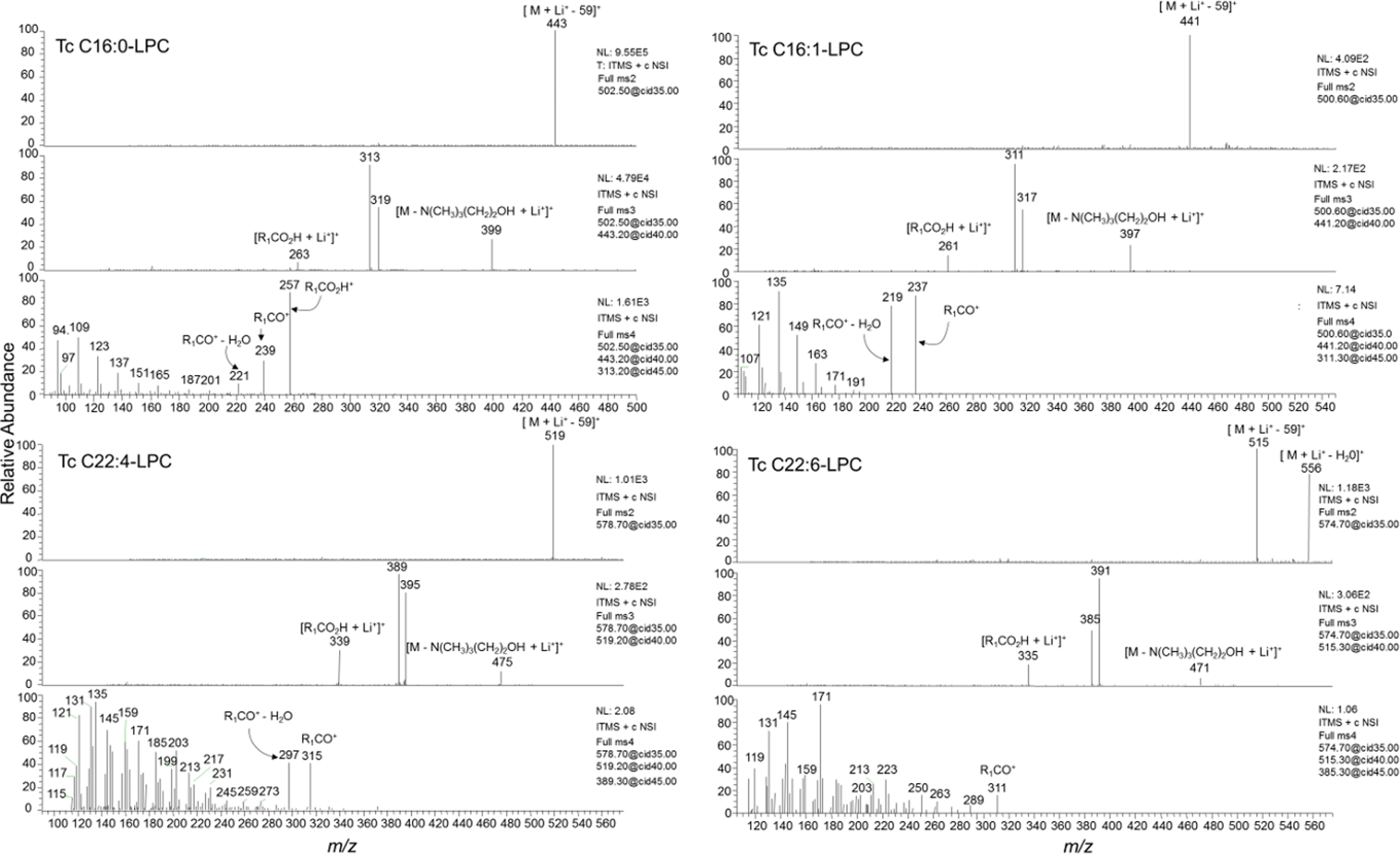

Supplement: Figure S4 — Tandem ESI-LIT-MS spectra of the low-abundance T. cruzi LPC-species enriched by POROS R1 fractionation. T. cruzi phospholipids from the 25% n-propanol fractions were diluted in methanol containing 5 mM LiOH and then infused directly into an LTQXL ESI-LIT-MS. Selected peaks were sequentially fragmented (MS2-MS4) by CID, as follows: C16:0-LPC (MS2 502→MS3 443→MS4 313); C16:1-LPC (MS2 500→MS3 441→MS4 311); C22:4-LPC (MS2 578→MS3 519→MS4 389); and C22:6-LPC (MS2 574→MS3 515→MS4 385). (TIF) [file pntd.0003077.s004.tif]

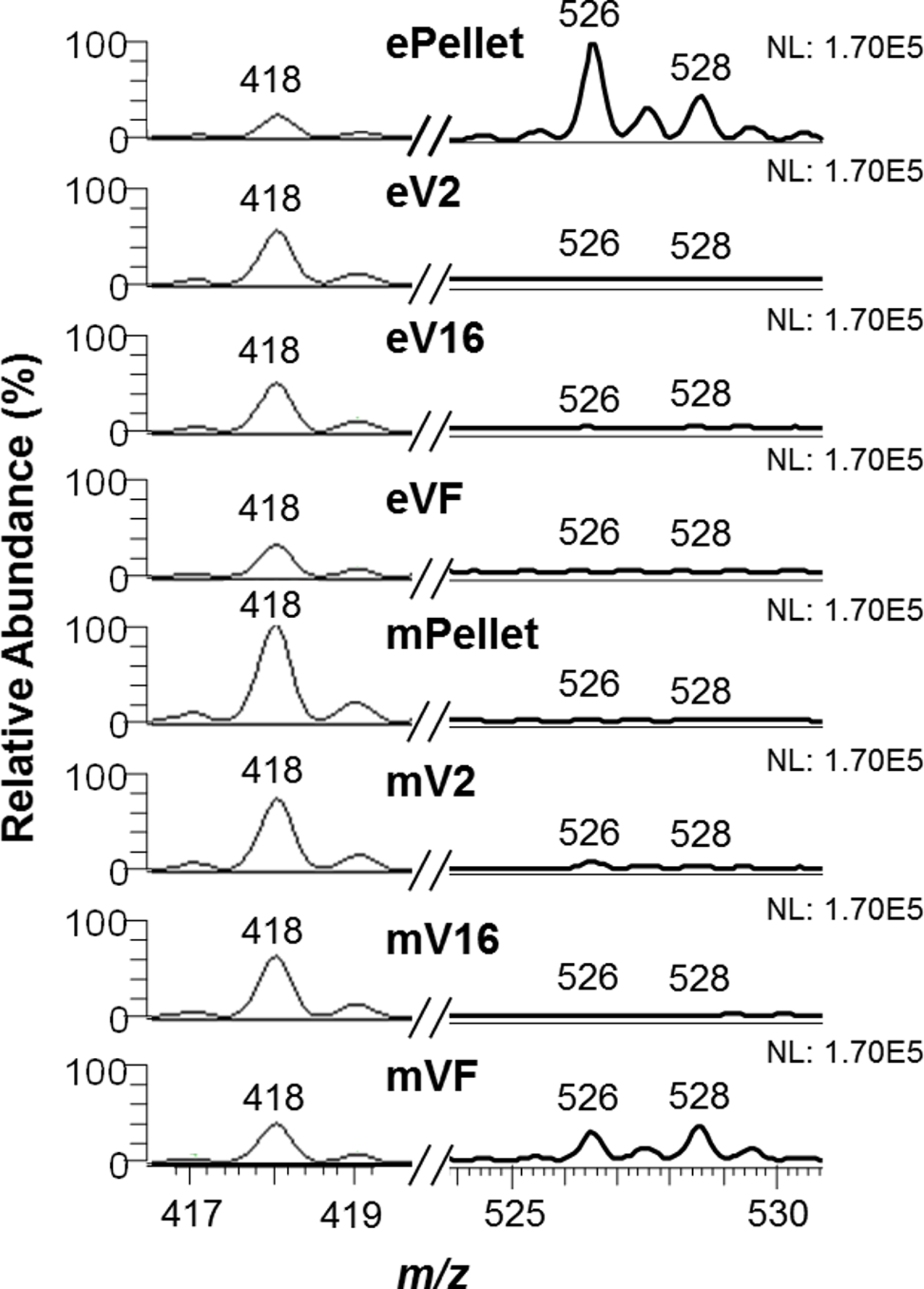

Supplement: Figure S5 — Quantification of LPC species in extracellular vesicles (EV) and EV-free supernatant of T. cruzi. C10:0-LPC (m/z 418) was used as an internal standard for quantification of the most abundant T. cruzi LPC species. C10:0-LPC was added to fresh pellets or EV preparations from Epis and Metas, prior to lipid extraction with C∶M (2∶1, v/v) and C∶M∶W (1∶2∶0.8, v/v/v), followed by Folch's partition. The Folch lower phase was analyzed by ESI-LIT-MS. C18:1- and C18:2-LPC are indicated at m/z 528 and 526, respectively. ePellet, Epi total pellet; eV2, Epi-derived ectosomes; eV16, Epi-derived exosomes; eVF, Epi-derived EV-free supernatant (or conditioned medium); mPellet, Meta total pellet; mV2, Meta-derived ectosomes; mV16, Meta-derived exosomes; mVF, Meta-derived EV-free supernatant. (TIF) [file pntd.0003077.s005.tif]

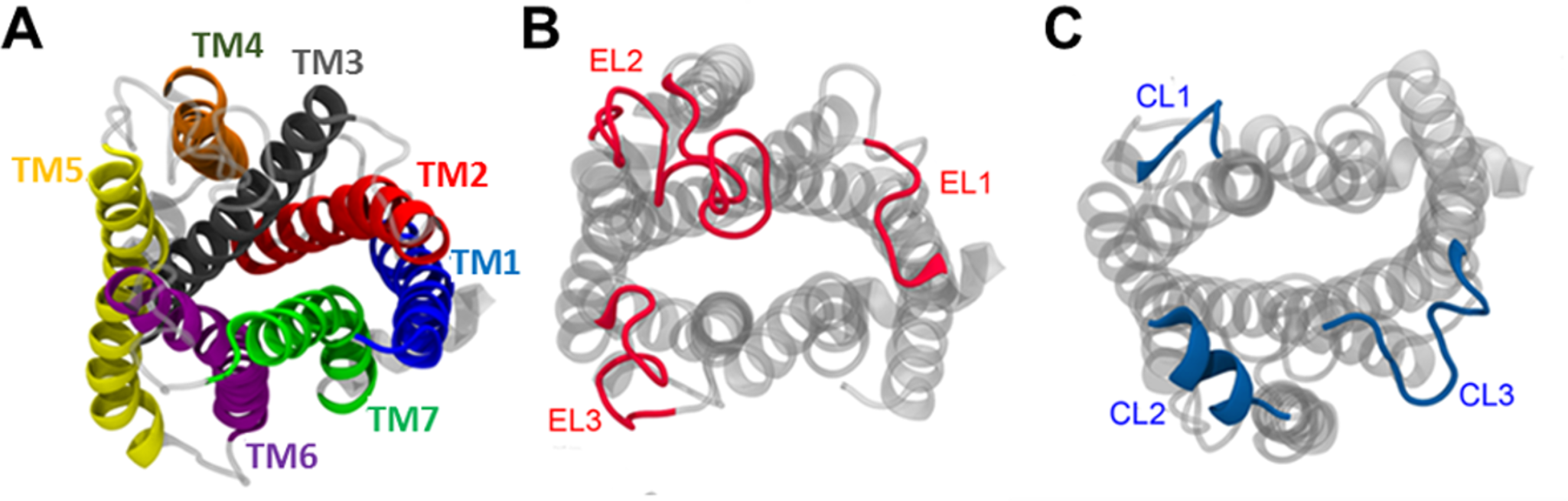

Supplement: Figure S6 — Structural representation of the PAFR model. (A) Arrangement of the 7 transmembrane (TM) helices forming a cavity. (B) The extracellular loops (EL1-EL3). (C) The intracellular loops (CL1-CL3). Each TM (alpha-helix) is indicated in a different color. TM1, blue; TM2, red; TM3, dark gray; TM4, orange; TM5, yellow; TM6, purple; TM7, green. (TIF) [file pntd.0003077.s006.tif]

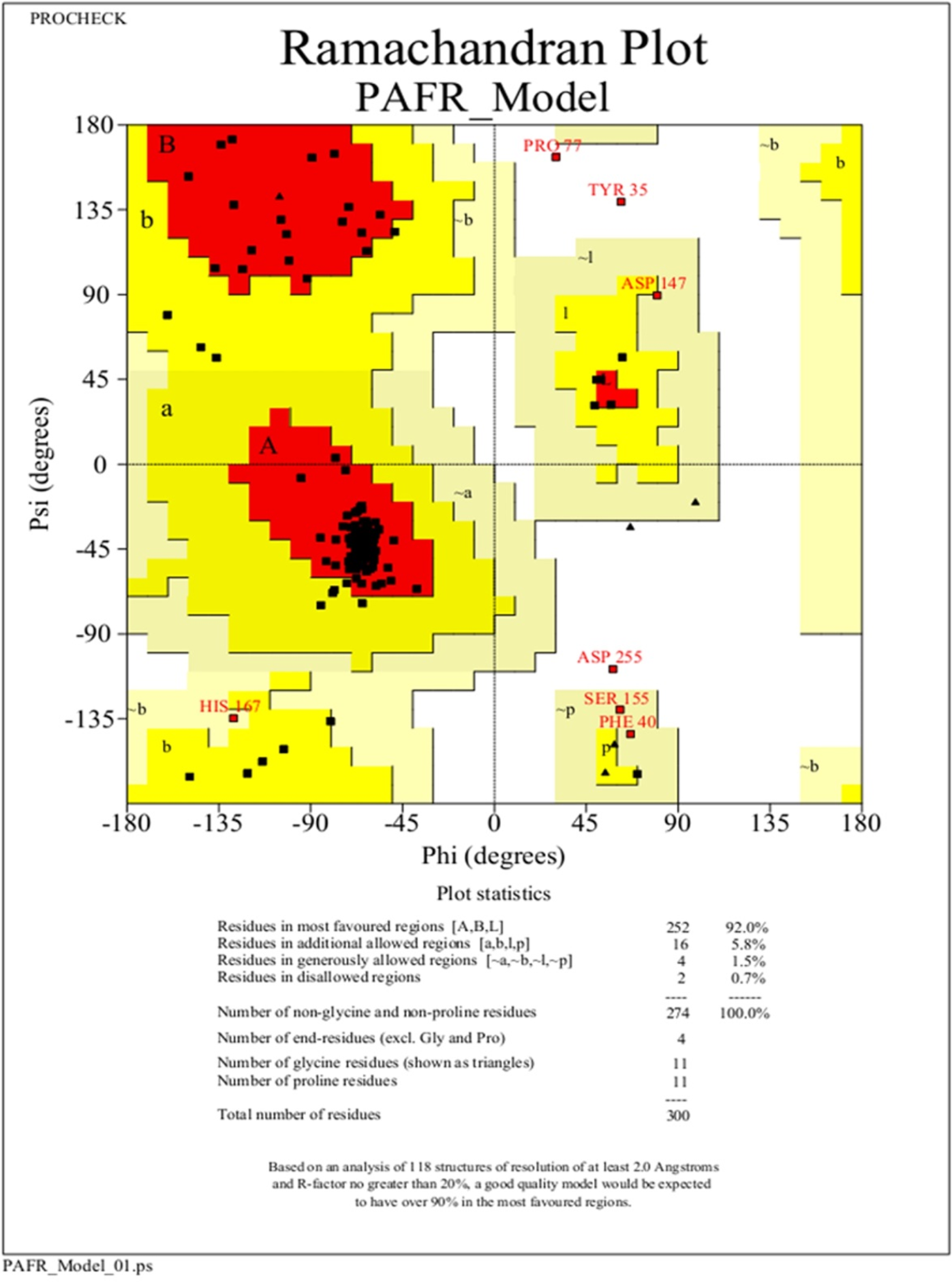

Supplement: Figure S7 — Main Ramachandran plot of the PAFR model. The main Ramachandran plot represents all amino acid residues by squares, whereas the glycine (Gly) residues are separately identified by black triangles, because these are not restricted to the regions in the generic Ramachandran plot. Red (A, B, and L) and dark yellow (a, b, l, and p) colors correspond to combinations of phi (Φ) and psi (Ψ) torsion angles of amino acid residues in the most favorable and additional allowed regions, respectively. In addition, pale yellow (∼a, ∼b, ∼l, and ∼p) and white colors represent less favorable and disallowed regions, respectively. The amino acid residues lying in those regions are highlighted in red. (TIF) [file pntd.0003077.s007.tif]

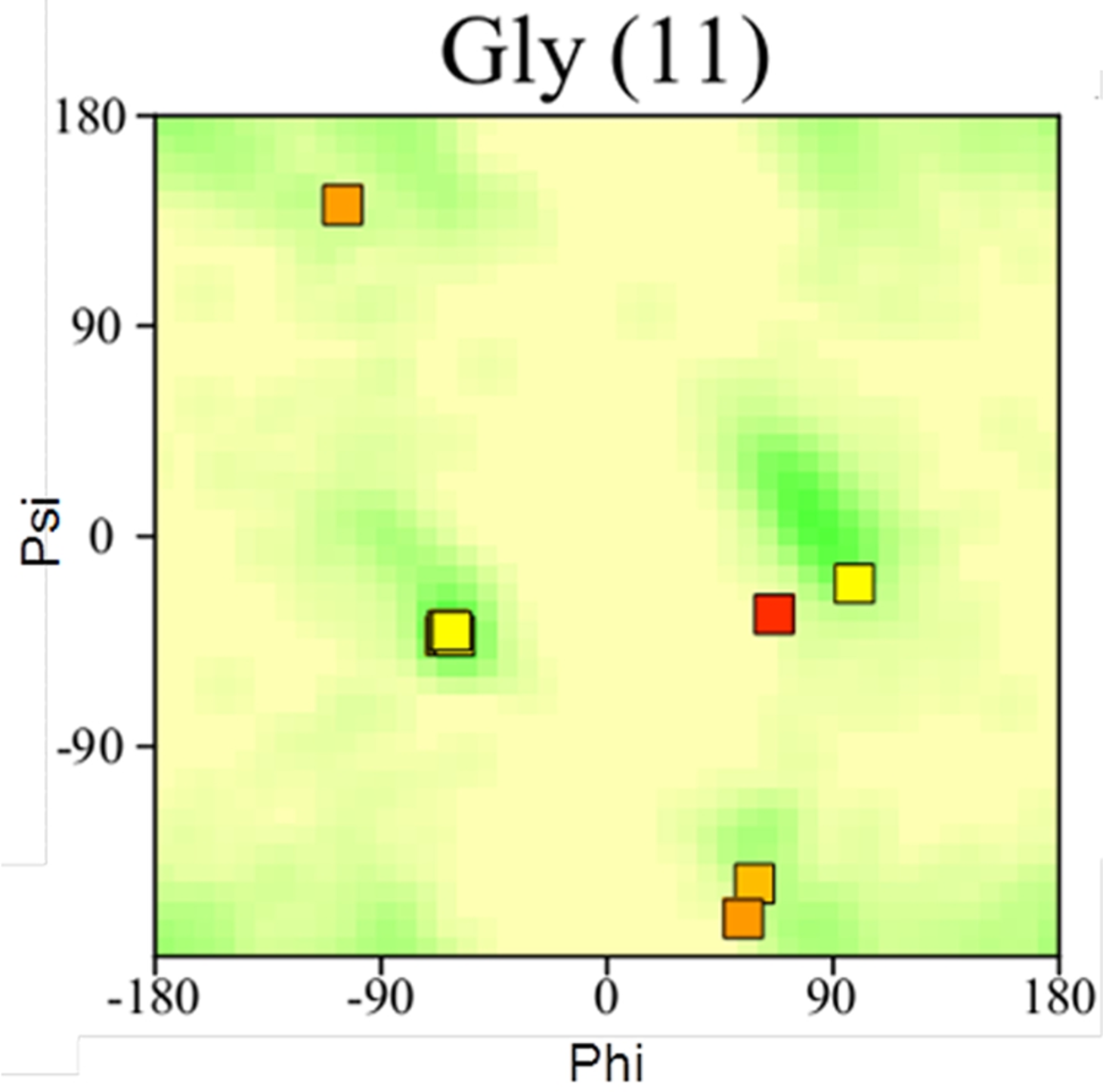

Supplement: Figure S8 — Ramachandran plot for Gly residues of the PAFR model. The second Ramachandran plot considers only Φ and ψ angles for Gly residues, where the number in parenthesis indicates the total number of these residues in the primary sequence of PAFR. The favorable combinations of these angles are represented by green areas and the values of standard deviations greater than 2.5 Å are labeled in red in the graph, describing combinations of disallowed angles. All Gly residues are in allowed regions, with favorable combinations of angles Φ and Ψ. (TIF) [file pntd.0003077.s008.tif]

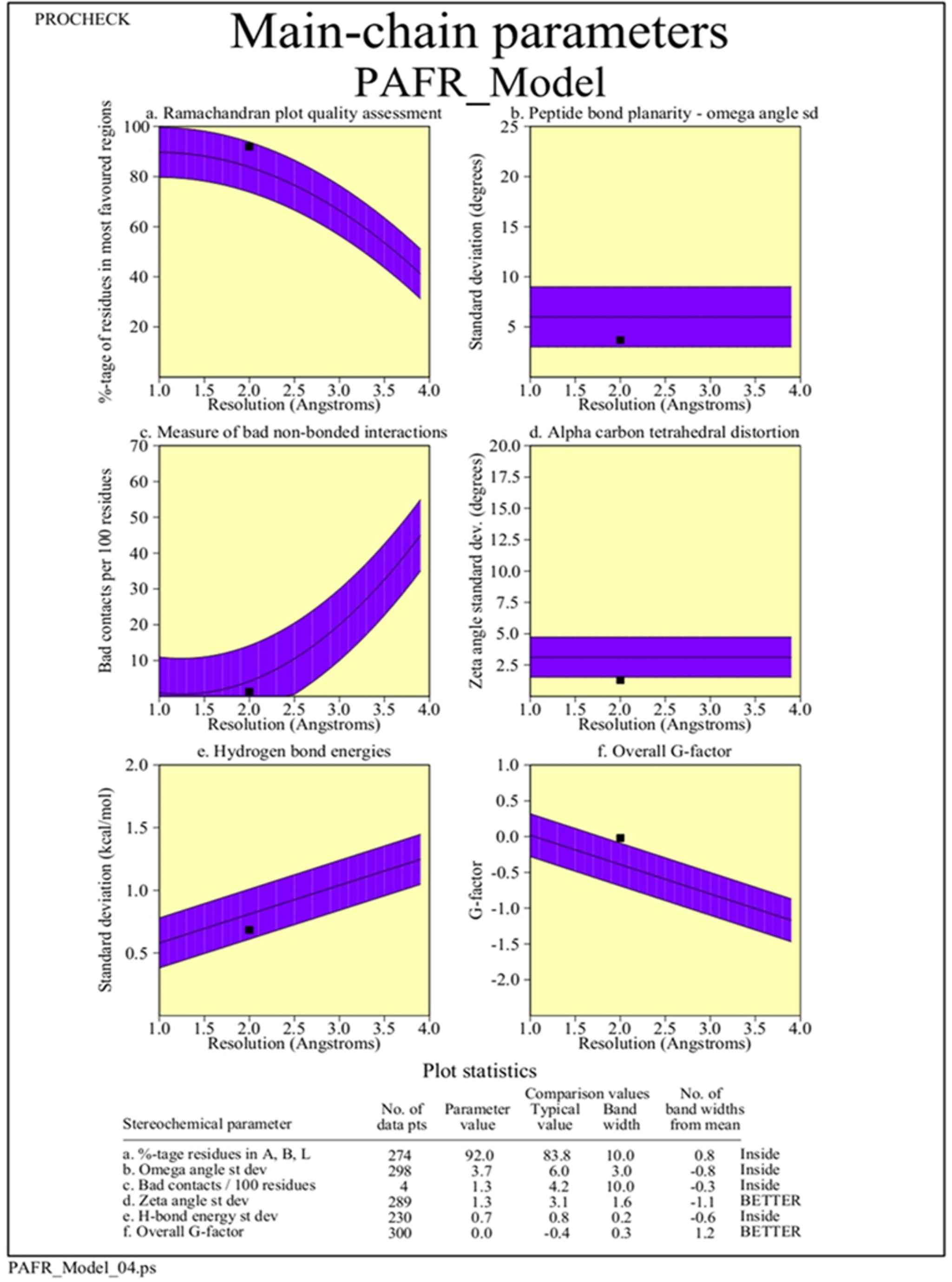

Supplement: Figure S9 — Analysis of the main-chain parameters of the PAFR model. The six graphs on the main chain parameters plot show how the PAFR model (represented by the black square) compares with a database of well-refined protein structures. The blue band in each graph represents the results from the well-refined protein structures; the central line is the least squares fitting to the mean trend as a function of resolution, whereas the width of the band on either side of it corresponds to a variation of one standard deviation from the mean. The six properties plotted are: (a) Ramachandran plot quality assessment; (b) peptide bond planarity; (c) number of bad contacts per 100 residues; (d) α-carbon tetrahedral distortion (this property is measured by calculating the standard deviation of the zeta torsion angle); (e) main chain hydrogen bond energy; and (f) overall G-factor (the overall value is obtained from an average of all the different G-factors for each residue in the protein structure). The G-factor provides a measure of how “normal”, or alternatively how “unusual”, a given stereochemical property is and, in PROCHECK, it is computed for the following properties: Φ-Ψ combination, chi1-chi2 (X1-X2; side chain torsion angles) combination, X1 torsion for those residues that do not have a X2, combined X3 and X4 torsion angles, omega torsion angles, main chain bond lengths, and main chain bond angles. (TIF) [file pntd.0003077.s009.tif]

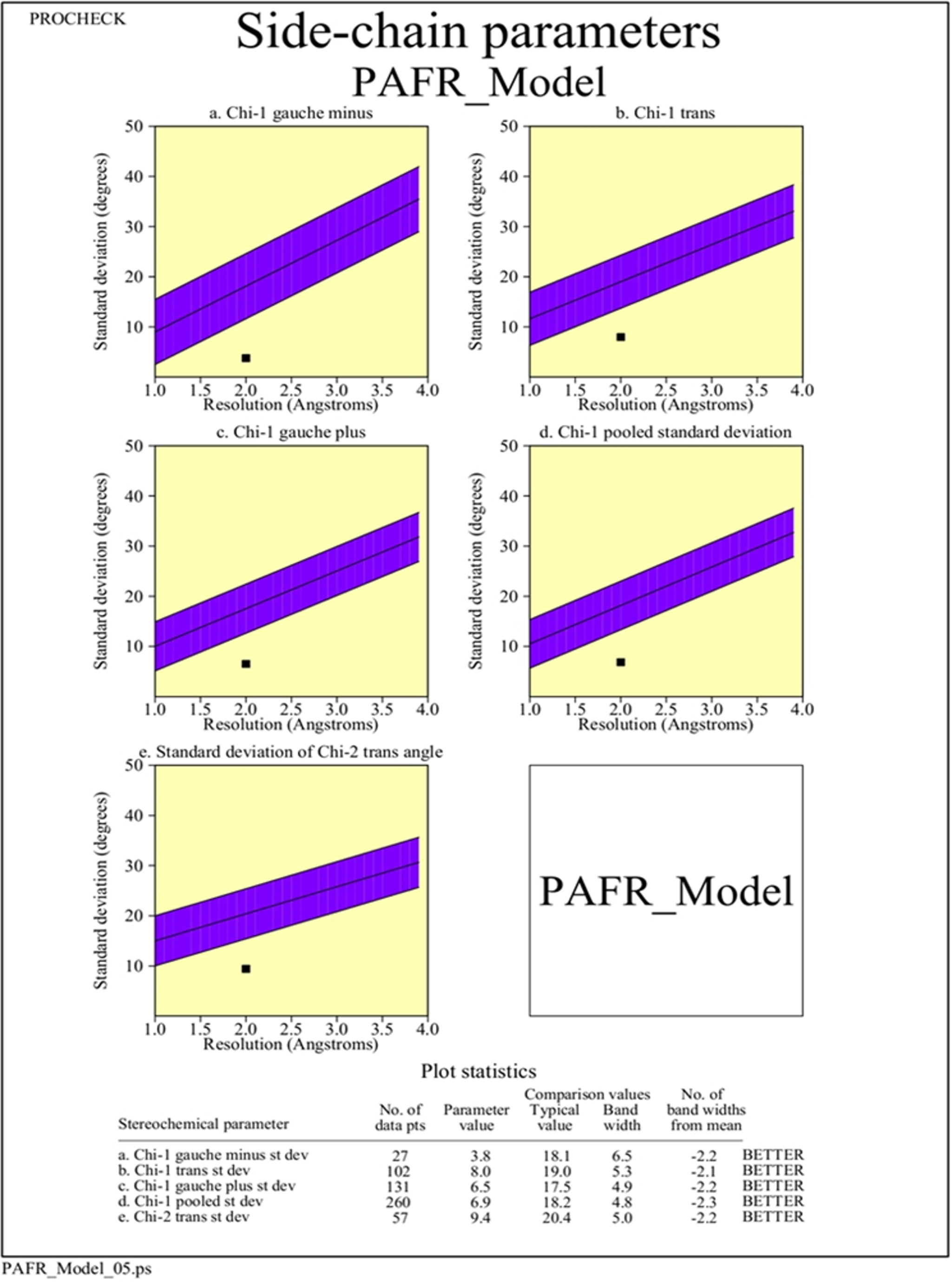

Supplement: Figure S10 — Analysis of the side-chain parameters of the PAFR model. In a similar way to the main-chain analysis, the side-chain analysis shows how the PAFR model (black square) compares with well-refined protein structures. This analysis evaluates five properties: (a) standard deviation values of the Chi-1 gauche minus torsion angles; (b) standard deviation values of the Chi-1 trans; (c) standard deviation values of the Chi-1 gauche plus; (d) pooled standard deviation of Chi-1 torsion angles; and (e) standard deviation values of the Chi-2 trans torsion angles. (TIF) [file pntd.0003077.s010.tif]

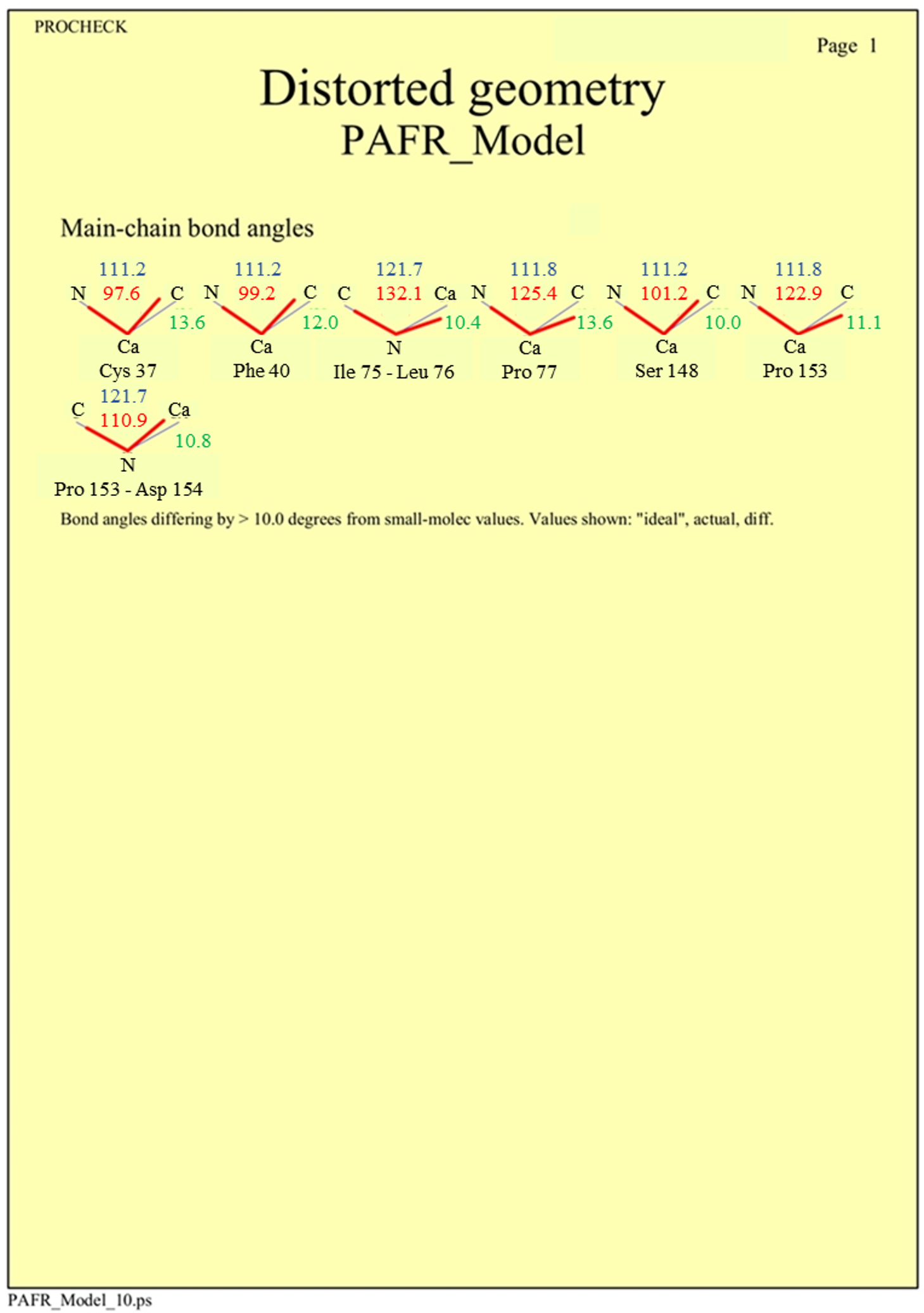

Supplement: Figure S11 — Analysis of geometrical distortions of the PAFR model. The parameters analyzed were: lengths and bond angles, including atoms of the main and side chains. This analysis shows the amino acid residues with distorted geometry, including their ideal values (in blue), those found in the model (in red), and the difference between these values (in green). (TIF) [file pntd.0003077.s011.tif]

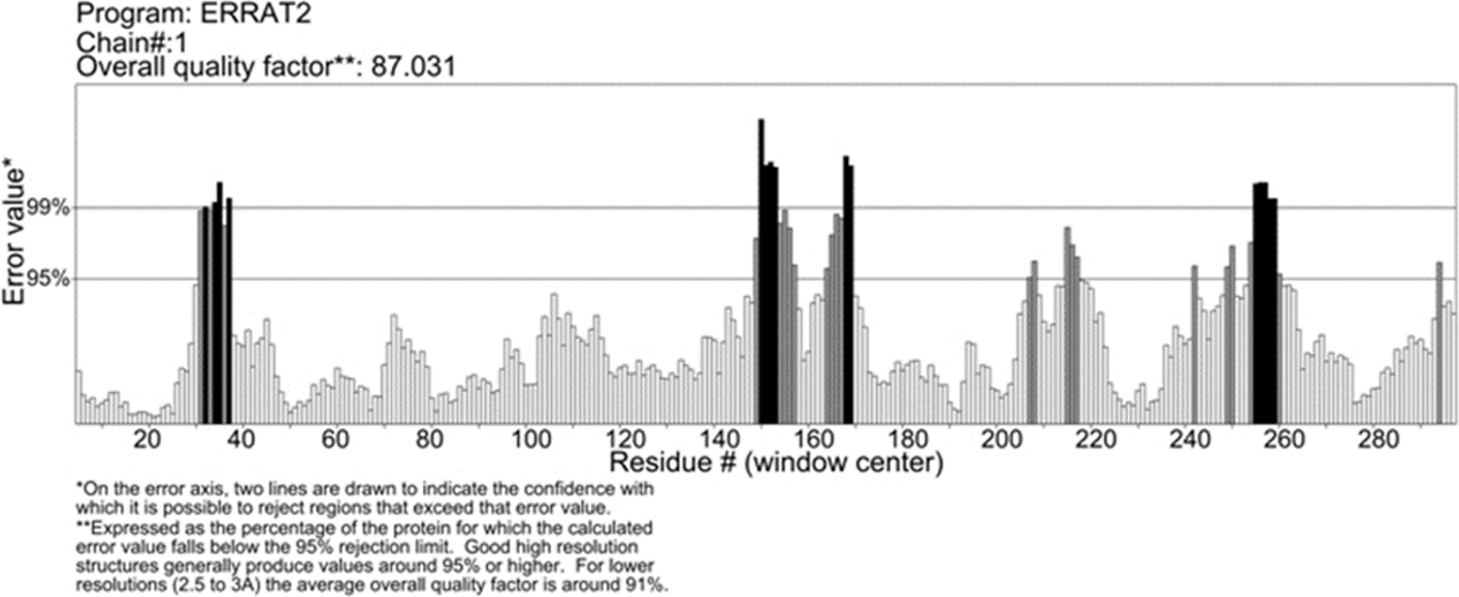

Supplement: Figure S12 — ERRAT analysis of the PAFR model. Errors in non-bonded atom-atom interactions of the PAFR model were verified by this analysis. The error values were plotted as a function of the position of a sliding residue in the window. An overall quality factor value of 87% was observed, thus validating the PAFR model. (TIF) [file pntd.0003077.s012.tif]

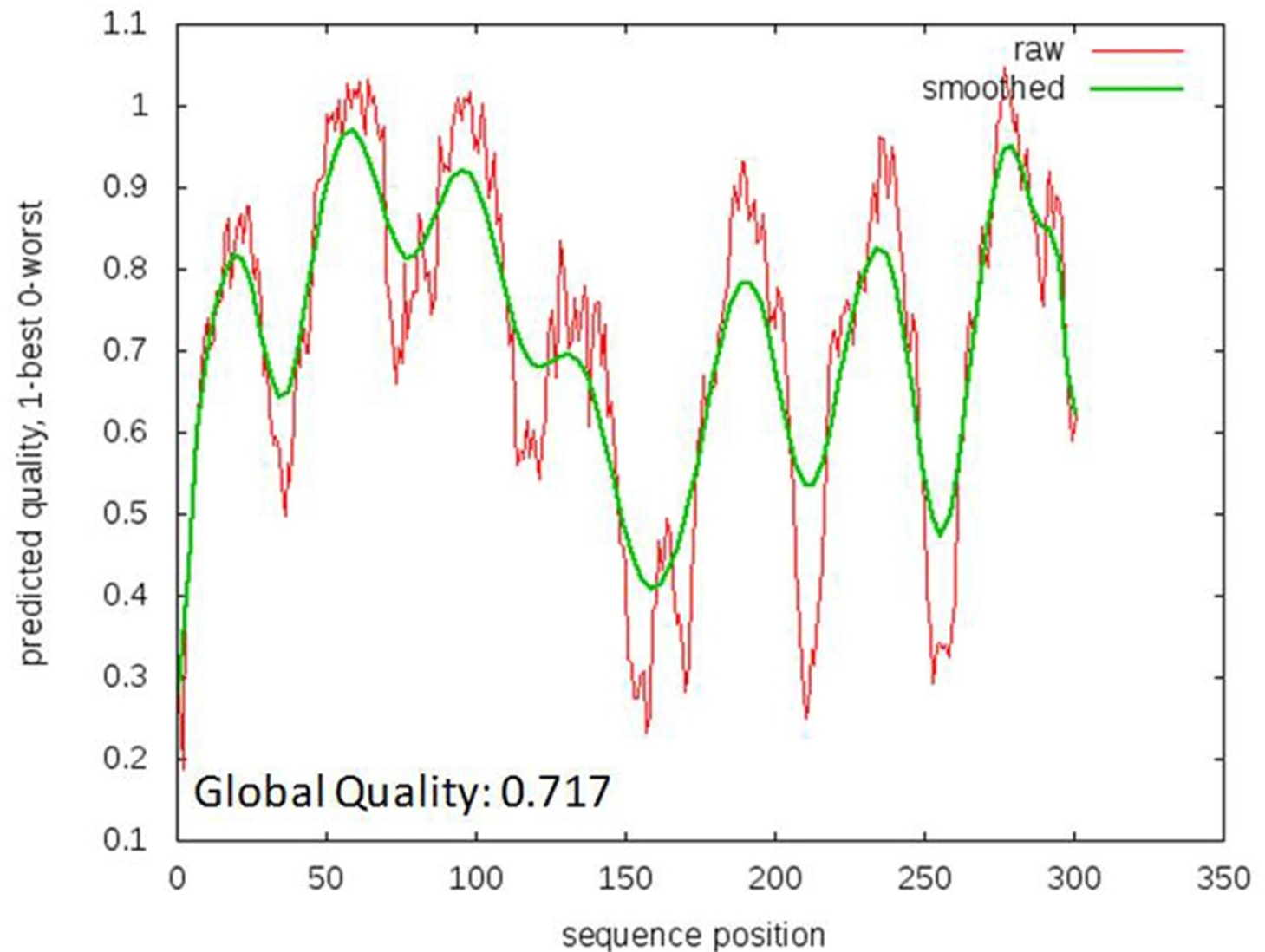

Supplement: Figure S13 — ProQM analysis of the PAFR model. A score was given to each residue of the protein model, which lead to a global quality factor of 0.717, which corroborates the other modelling analyses. (TIF) [file pntd.0003077.s013.tif]

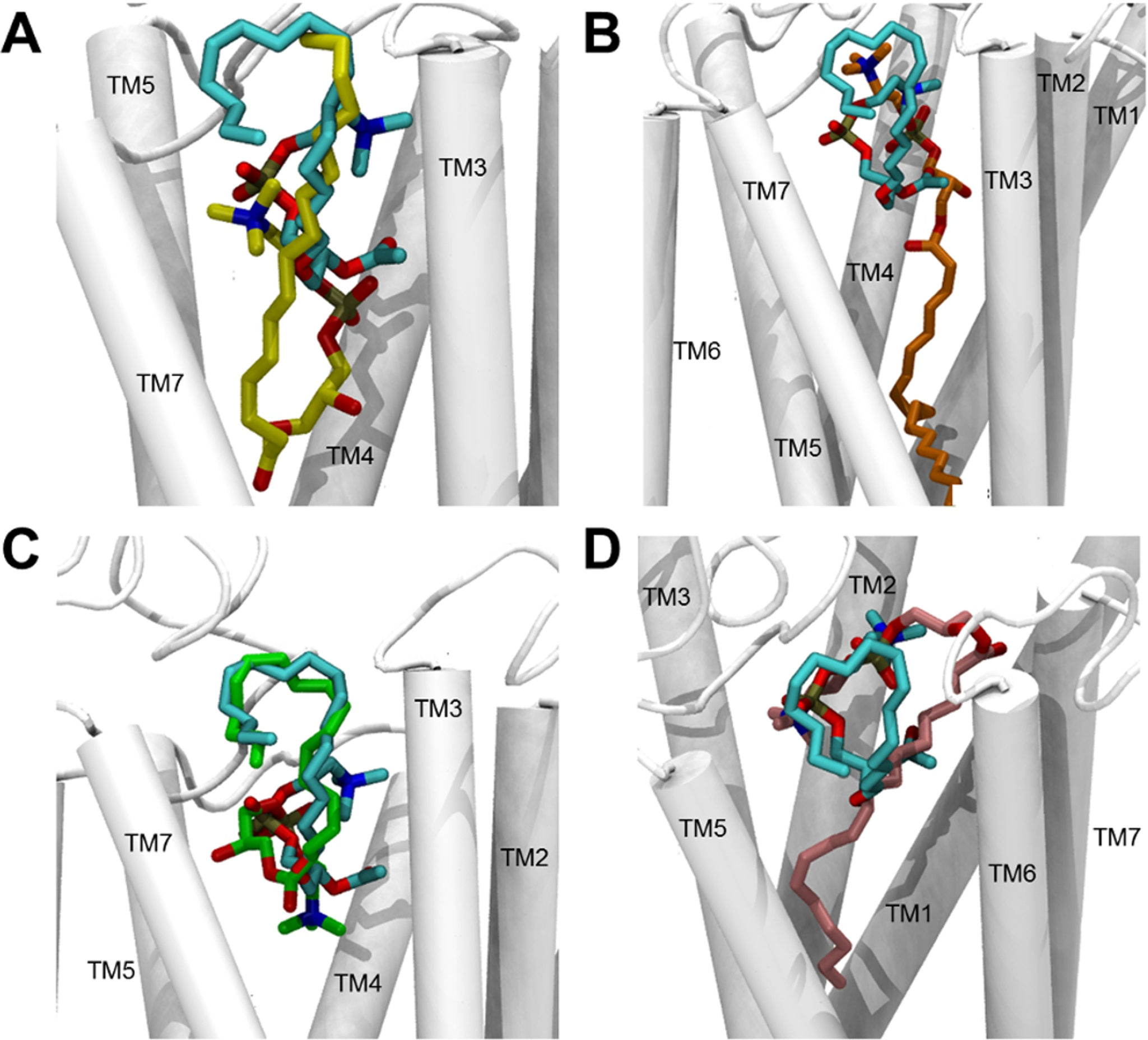

Supplement: Figure S14 — Comparison of binding modes of PAF and each LPC species to PAFR. (A) C16:0-PAF (cyan) and C16:0-LPC (yellow); (B) C16:0-PAF (cyan) and C18:0-LPC (orange); (C) C16:0-PAF (cyan) and C18:1-LPC (green); (D) C16:0-PAF (cyan), and C18:2-LPC (gray). Transmembrane (TM) regions are represented as white rods. (TIF) [file pntd.0003077.s014.tif]

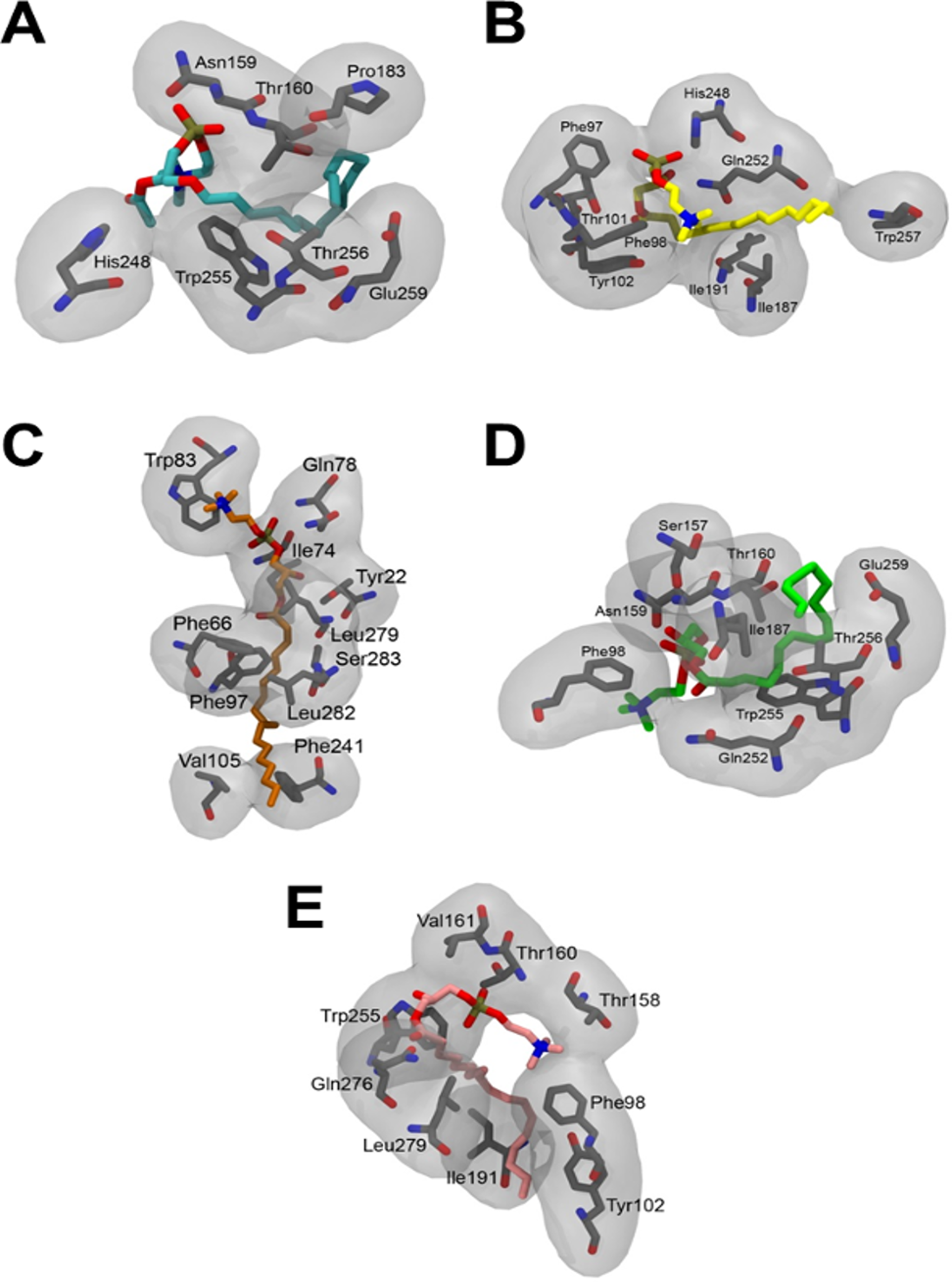

Supplement: Figure S15 — Unfavorable steric interactions of PAF and LPCs with PAFR. Heteroatoms are represented by different colors in structures: nitrogen atoms are shown in blue, oxygen in red, and carbon atoms of amino acids in gray. In ligands, carbon chains are represented by different colors: (A) C16:0-PAF, cyan; (B) C16:0-LPC, yellow; (C) C18:0-LPC, orange; (D) C18:1-LPC, green; and (E) C18:2-LPC, pink. (TIF) [file pntd.0003077.s015.tif]
