# Supplementary material for: Structural and Functional Analysis of a Platelet-Activating Lysophosphatidylcholine of Trypanosoma cruzi
Source: PLoS Negl Trop Dis. 2014 Aug 7;8(8):e3077. doi: 10.1371/journal.pntd.0003077 (PMC4125143; doi:10.1371/journal.pntd.0003077)
Supplement: Table S1 — Quantification of LPC species in extracellular vesicles (EVs) and EV-free supernatant of epimastigotes and metacyclic trypomastigote forms. (DOCX) [file pntd.0003077.s016.docx]

| **LPC species** | **Picomoles of LPC per 10^6^ cells** | **LPC amount**  **(mol^-17^) per parasite *^a, b^*** | **Number of LPC**  **molecules per parasite *^c^*** |
| --- | --- | --- | --- |
| **C18:2-LPC (*m/z* 526.4)** | | | |
| ePellet *^d^* | **2.7** | 0.36 | 2.16 x 10^6^ |
| eV2 | **n/a** *^e^* | **n/a** | **n/a** |
| eV16 | 0.9 | 0.12 | 0.72 x 10^6^ |
| eVF | 0.7 | 0.09 | 0.54 x 10^6^ |
| mPellet | 0.6 | 0.08 | 0.48 x 10^6^ |
| mV2 | 0.8 | 0.11 | 0.66 x 10^6^ |
| mV16 | **n/a** | **n/a** | **n/a** |
| mVF | **1.8** | 0.24 | 1.44 x 10^6^ |
| **C18:1-LPC (*m/z* 528.4)** | | | |
| ePellet | **1.3** | 0.18 | 1.08 x 10^6^ |
| eV2 | **n/a** | **n/a** | **n/a** |
| eV16 | 1.0 | 0.14 | 0.81 x 10^6^ |
| eVF | 0.7 | 0.09 | 0.52 x 10^6^ |
| mPellet | 0.3 | 0.05 | 0.30 x 10^6^ |
| mV2 | 0.5 | 0.07 | 0.43 x 10^6^ |
| mV16 | **n/a** | **n/a** | **n/a** |
| mVF | **1.9** | 0.25 | 1.5 x 10^6^ |

**Table S1.** Quantification of LPC species in extracellular vesicles (EVs) and EV-free supernatant of epimastigotes and metacyclic trypomastigote forms.

*^a^* The molar relative response factors (MRRF) of C10:0-LPC and LPC standards were used to calculate the amount of each LPC molecular species in Folch lower-phase fractions of *T. cruzi* .

*^b^* The number of parasites was determined before lipid extraction by counting live parasites in a hemocytometer. Values are means of three determinations. The standard deviation in all cases was <15%.

*^c^* Determined by multiplying the number of moles by the Avogadro’s constant. D

*^d^* ePellet, Epi pellet; eV2, Epi-derived ectosomes; eV16, Epi-derived exosomes; eVF, Epi-derived EV-free supernatant or fraction; mPellet, Meta pellet; mV2, Meta-derived ectosomes; mV16, Meta-derived exosomes; mVF, Meta-derived EV-free supernatant or fraction.

*^e^* Not analyzed due to absence or trace amounts of the compound.
